# The role of gut microbiota in shaping the relapse-remitting and chronic-progressive forms of multiple sclerosis in mouse models

**DOI:** 10.1038/s41598-019-43356-7

**Published:** 2019-05-06

**Authors:** K. Alexa Orr Gandy, Jiajia Zhang, Prakash Nagarkatti, Mitzi Nagarkatti

**Affiliations:** 1 Department of Pathology, Microbiology and Immunology, University of South Carolina, School of Medicine, Columbia, USA; 2 Epidemiology and Biostatistics, Arnold School of Public Health, University of South Carolina, Columbia, USA; 3 WJB Dorn VA Medical Center, 29208 Columbia, SC USA

**Keywords:** Autoimmunity, Autoimmunity, Autoimmunity, Microbiome, Microbiome

## Abstract

Using a mouse model of multiple sclerosis (MS), experimental autoimmune encephalitis (EAE), we evaluated the role of gut microbiota in modulating chronic-progressive (CP) versus relapse-remitting (RR) forms of the disease. We hypothesized that clinical courses of EAE may be shaped by differential gut microbiota. Metagenomic sequencing of prokaryotic 16S rRNA present in feces from naïve mice and those exhibiting CP-EAE or RR-EAE revealed significantly diverse microbial populations. Microbiota composition was considerably different between naïve strains of mice, suggesting microbial components present in homeostatic conditions may prime mice for divergent courses of disease. Additionally, there were differentially abundant bacteria in CP and RR forms of EAE, indicating a potential role for gut microbiota in shaping tolerant or remittance-favoring, and pathogenic or pro-inflammatory-promoting conditions. Furthermore, immunization to induce EAE led to significant alterations in gut microbiota, some were shared between disease courses and others were course-specific, supporting a role for gut microbial composition in EAE pathogenesis. Moreover, using Linear Discriminant Analysis (LDA) coupled with effect size measurement (LEfSe) to analyze microbial content, biomarkers of each naïve and disease states were identified. Our findings demonstrate for the first time that gut microbiota may determine the susceptibility to CP or RR forms of EAE.

## Introduction

Experimental autoimmune encephalomyelitis (EAE) is a well-established mouse model of the neurodegenerative disease multiple sclerosis (MS) and is characterized by CD4+ T cell-mediated autoimmune destruction of protective myelin sheaths surrounding neurons in the central nervous system (CNS)^1^. Patients with MS exhibit a wide range of severity and duration of symptoms, as well as extent of neurological damage. In humans, the relapse-remitting form of MS (RRMS) is the most common subtype of disease and is distinguished by acute inflammatory flare-ups followed by periods of remission^2^. Ultimately due to accumulation of neurological damage, many people with RRMS go on to develop the secondary progressive (SPMS) form of the disease whereby they experience progressive neurologic decline and remission between attacks becomes less defined^2^. In the second sub-type seen in a small proportion of patients, called the primary progressive form (PPMS), there is a steady increase in disability, in the absence of acute attacks, and without periods of remission^2^. There are many different laboratory mouse models of MS, each having a unique contribution to gaining a better understanding of the human disease^3^. One, induced by immunization with myelin oligodendrocyte peptide (MOG_35–55_), in the C57BL/6 strain of mouse, mimics PPMS with a chronic-progressive course of disease (CP-EAE). Secondly, immunization with myelin proteolipid protein peptide (PLP_139–151_), in the SJL/J strain of mouse, leads to a relapse-remitting course of disease (RR-EAE), similar to RRMS^4^. It is unclear why these two strains of mice exhibit differing disease courses; however, it is generally believed that this may result from the differential induction of pro-inflammatory Th1/Th17 cells versus anti-inflammatory Tregs^5^. While there are many rodent models of MS, including transgenic mouse models that experience spontaneous EAE and induced EAE in non-obese diabetic (NOD) mice, studies evaluating the contribution of genetic strain on shaping disease pathogenesis and progression are lacking^3,6^.

While the etiology of MS remains unclear, a combination of factors is thought to contribute: immunologic, environmental, infectious and genetic^7,8^. Recently, studies have indicated gut microbial composition as a critical environmental component of autoimmune disease pathogenesis. Commensal gut microbes have been shown to affect host metabolic functions as well as host immune functions, as gut microbes are involved in host formation of gut-associated lymphoid tissue, and CD4+ T helper cell differentiation and balance, and therefore play an important role in shaping the overall immune response^9–11^. Specifically in EAE, germ-free mice and mice treated with oral antibiotics exhibit decreased disease severity, which is associated with an increase in immune tolerance via generation of Tregs^12,13^. Furthermore, specific types of bacteria or bacterial products can alter EAE in rodents; for example, mice colonized with segmented filamentous bacteria (SFB) exhibit more severe disease symptoms due to generation of pro-inflammatory Th17 cells^13^. On the contrary, when exposed to Polysaccharide A (PSA), a capsular polysaccharide from gut commensal *Bacteroides fragilis*, mice are protected from EAE as a result of potentiation of pro-inflammatory Th1 activity and promotion of anti-inflammatory Treg activity^14^. Moreover, microbial dysbiosis has been associated with EAE susceptibility and resistance in rodents, as well as observed in patients with MS^15–19^. Most recently, for example, our lab identified a role for gut microbial composition in mediating the protective effects of CD44 knockout (CD44KO) on EAE pathogenesis. CD44KO mice exhibit a shift from pro-inflammatory, pathogenic Th17 cells to anti-inflammatory, tolerance-inducing Treg cells and this is dependent on gut microbiota, as transfer of fecal material form CD44KO mice but not CD44 wild-type mice led to significant amelioration of EAE^20^. While other models, including transgenic models and the NOD model, of EAE have been used to evaluate the impact of gut microbiota on the pathogenesis and progression of the disease, a direct evaluation of the role of microbiota shaped by mouse strain on disease has not been carried out^3,6^.

In the current study, we hypothesized that differential susceptibility to EAE and the distinct clinical forms of the disease seen in SJL/J and C57BL/6 mice may result from differences in gut microbial composition in these strains and that encephalitogenic immunization would cause dysbiosis of the gut microbiome with unique bacteria emerging that influence the disease. The current study provides support to this hypothesis and indicates a possible role for gut microbiota in the regulation of clinical forms of EAE.

## Results

### Core Diversity

**Figure 1:**
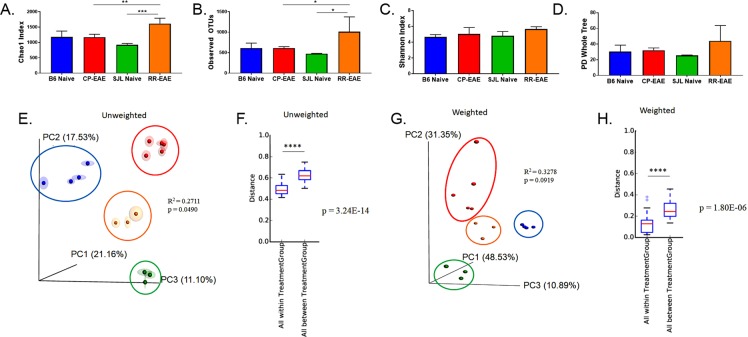
Diversity of fecal microbiota. Stool samples were collected from naïve C57BL/6 (n = 4) and SJL/J (n = 3) mice or from mice exhibiting the chronic progressive (CP-EAE) (n = 5) or relapse-remitting (RR-EAE) (n = 3) EAE disease course at the initial peak of disease (day 13 post immunization) and microbial composition determined. Evaluation of microbial richness and diversity within each group (α-diversity) was assessed using the (**A**) *Chao1* index (Naïve SJL/J vs RR-EAE p = 0.0004; CP-EAE vs RR-EAE p = 0.0052), (**B**) distinct number of OTUs observed (Naïve SJL/J vs RR-EAE, p = 0.0115; CP-EAE vs RR-EAE, p = 0.0351) (**C**) Shannon Index, and (**D**) Phylogenetic Diversity measure (PD Whole Tree). One-way ANOVA with Tukey’s post hoc analysis was used to generated the reported, adjusted p-values. Principle coordinate analysis (PCoA) plots were generated from (**E**) Unweighted and (**G**) Weighted UniFrac distance metrics to assess similarity between groups (β-diversity). PERMANOVA analysis was assessed using the adonis function in the vegan package of R (Unweighted p = 0.049, R^2^ = 0.2711; Weighted p = 0.0919, R^2^ = 0.3278). Distance boxplots of (**F**) Unweighted and (**H**) Weighted UniFrac metrics were used to assess diversity within and between groups. Pairwise comparison amongst “within all groups” and “between all groups” was performed using two-sample t-tests with Bonferroni correction. P-values reported are adjusted values (Unweighted, p = 3.24E-14; Weighted, p = 1.80E-06). Output was generated using the Qiime pipeline within NIH-supported microbiome analysis software Nephele.

In order to evaluate the role of the gut microbiome in initiating divergent courses of EAE, 8–10-week-old female C57BL/6 or SJL/J mice were immunized with MOG or PLP, respectively. Mice were evaluated daily for clinical symptoms of disease. At the peak of disease, stool was collected from mice exhibiting the chronic progressive (CP-EAE; MOG-induced EAE in C57BL/6 strain) or relapse remitting (RR-EAE; PLP-induced EAE in SJL/J strain) form of EAE, or from naïve mice (C57BL/6 or SJL/J). Both strains of mice exhibited an initial peak in disease severity on day 13 post -immunization; however, while mice with the CP form of the disease continued to progress over time, mice with the RR form of the disease remitted on day 16, which was followed by an acute relapse on day 17, an additional remittance at day 25, and relapse again at day 31, where a change in disease severity score of ±1 was used to define a relapse or remittance (Supplemental Fig. S1)^4^. The two courses of disease were found to be significantly different using the Mann-Whitney test (p = 0.0241). Stool was taken on day 13 at the initial peak of disease, when the scores of CP- and RR-EAE mice were the same (Supplemental Fig. S2A). This was done in order to evaluate the role the microbiota may play in initiating divergent courses of disease, specifically in the initial remittance observed in RR-EAE mice (Supplemental Fig. S1). Furthermore, in line with previous studies, the expression of FoxP3 in encephalitogenic T-cells from RR-EAE mice was significantly higher than in T cells from the brains of CP-EAE mice at the peak of disease, indicating a more immune-tolerant environment, which has been shown to coincide with and drive episodes of remittance in both human MS and mouse models^21^ (Supplemental Fig. S2B). Next, metagenomic sequencing of the variable V4 region of the prokaryotic 16S ribosomal RNA gene present in feces was performed. Sequencing analysis revealed significantly diverse microbial populations in the CP and RR forms of EAE, as well as in the two strains of naïve mice. Bacterial richness and evenness within each group (α-diversity) was estimated using the *Chao1* index, observed OTUs, phylogenetic diversity whole tree measurements (PD whole tree), and Shannon entropy (Fig. 1A–D). Differences in α-diversity metrics were assessed using a one-way ANOVA with Tukey’s post hoc analysis and adjusted p-values are reported in Table 1. *Chao1* and observed OTUs were used to assess differences in richness, and while there were no significant differences observed in naïve groups relative to one-another or in naïve C57BL/6 relative to CP-EAE, there were statistically significant differences in richness between the two diseased groups (*Chao1*, p = 0.0052; Obs OTUs, p = 0.0351), as well as between RR-EAE and naive SJL/J groups (*Chao1*, p = 0.0004; Obs OTUs, p = 0.0115) (Fig. 1A,B and Table 1). Furthermore, there were no significant differences detected between groups using PD whole tree and Shannon indices as metrics (Fig. 1C,D and Table 1).

**Table 1:** Alpha-Diversity Metrics. Mean values of indicated metrics are reported.

		B6 Naïve	CP-EAE	SJL/J Naïve	RR-EAE	p-value
Richness	Chao1	1179.27		919.47		0.1231
		1179.27	1173.22			0.9999
				919.47	1616.30	0.0004
			1173.22		1616.30	0.0052**
	Observed Speices	610.50		475.93		0.7249
		610.50	614.12			>0.9999
				475.93	1011.57	0.0115*
			614.12		1011.57	0.0351*
Diversity	PD Whole Tree	30.42		25.60		0.9124
		30.42	32.26			0.9914
				25.60	44.08	0.1467
			32.26		44.08	0.3800
	Shannon	4.67		4.80		0.9893
		4.67	5.05			0.7608
				4.80	5.68	0.3071
			5.05		5.68	0.4836

One-way ANOVA with multiple comparisons was used to assess significance. Tukey’s post-test was performed to generate adjusted p-values reported here. **p < 0.01; *p < 0.05.

In order to evaluate the diversity between groups (β-diversity), weighted and unweighted UniFrac metrics were used to generate 3D principle coordinate analysis (PCoA) plots. Using the presence of OTUs, while also taking into account the phylogenetic distances between observed OTUs, the unweighted UniFrac metrics were transformed into a PCoA plot where the principle component scores accounted for 21.2% (PC1), 17.5% (PC2) and 11.1% (PC3) of the total variation. Moreover, sample clustering within each treatment group was clearly defined (Fig. 1E). Furthermore, using the abundance of OTUs, in addition to phylogenetic distances, weighted UniFrac calculations were used to generate a PCoA plot, where principle component scores accounted for 48.5% (PC1), 31.4% (PC2) and 10.9% (PC3) of the total variation with a clear delineation between sample clustering within each treatment group (Fig. 1G). Additionally, permutational multivariate analysis of variance (PERMANOVA) using Qiime-generated UniFrac weighted and unweighted distance matrices was carried out with the adonis function in R vegan package using 1000 permutations. While PERMANOVA analysis of unweighted UniFrac distances between groups was significant, with 27.1% of the proportion of variation explained by grouping (p = 0.049, R^2^ = 0. 2711), analysis of weighted UniFrac distances was not significant (p = 0.0919, R^2^ = 0.3278). Furthermore, in order to evaluate within-group and between-group distances and assess significance, distance boxplots were generated and significance was assessed by two-sample t-tests for all pairs of boxplots, using Bonferroni correction to generate adjusted p-values (Supplemental Tables S3 and S4). With both unweighted and weighted UniFrac distances, a significant difference between the ‘within all treatment groups” and “between all treatment groups” was identified, indicating that samples within each group are significantly more similar to each other than samples between groups (Unweighted, p = 3.24E-14; Weighted, p = 1.80E-06) (Fig. 1F,H and Supplemental Tables S3 and S4).

### Analysis of Microbial Composition

**Figure 2:**
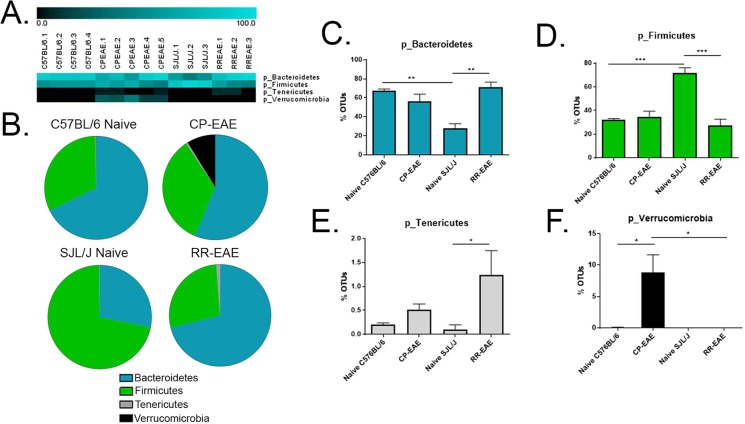
Fecal microbial composition at the Phylum Level. Stool samples were collected as described in Fig. 1 legend and microbial composition determined by 16 S rRNA V4 hypervariable sequencing using Illumina MiSeq System. (**A**) Heat maps represent percent total Operational Taxonomic Units (OTUs) identified in each sample (Naïve C57BL/6; C57BL/6.1-C57BL/6.4) (n = 4); (C57BL/6 EAE; CPEAE.1-CPEAE.5) (n = 5); (Naïve SJL/J; SJL/J.1-SJL/J.3) (n = 3) and (SJL/J EAE; RREAE.1-RREAE.3) (n = 3) and were generated using Genesis software. (**B**) Mean percent OTU abundance represented as pie chart for each group. (**C**–**F**) One-way ANOVA, followed by Tukey’s multiple comparisons, was performed in order to assess significance in the indicated phyla. Bars represent mean ± SEM and exact p-values indicated in text (*p < 0.05; **p < 0.01; ***p < 0.001; ****p < 0.0001).

**Figure 3:**
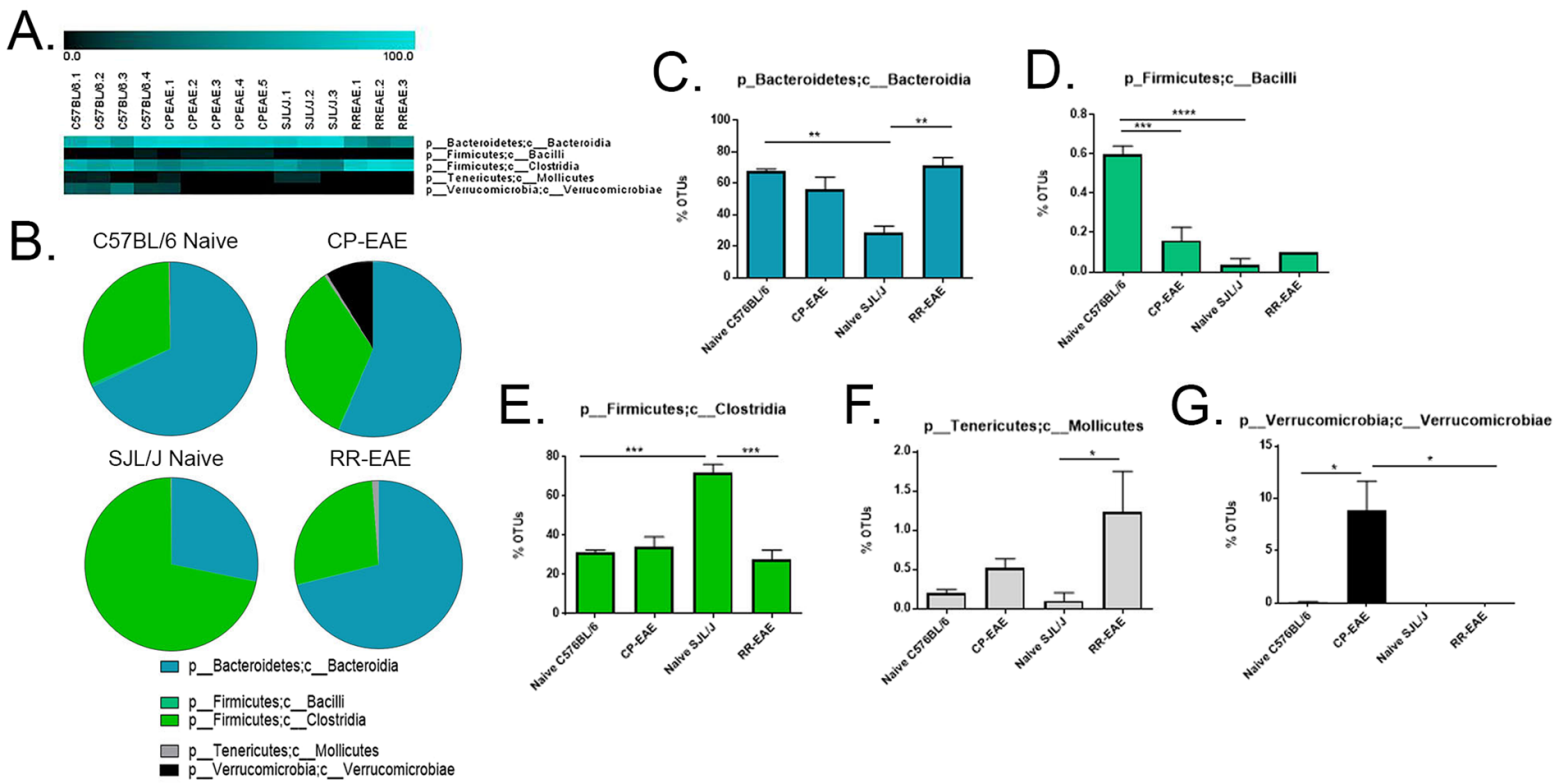
Fecal microbial composition at the Class Level. Stool samples were collected as described in Fig. 1 legend and microbial composition determined by 16S rRNA V4 hypervariable sequencing using Illumina MiSeq System. (**A**) Heat maps represent percent total Operational Taxonomic Units (OTUs) identified in each sample (Naïve C57BL/6; C57BL/6.1-C57BL/6.4) (n = 4); (C57BL/6 EAE; CPEAE.1-CPEAE.5) (n = 5); (Naïve SJL/J; SJL/J.1-SJL/J.3) (n = 3) and (SJL/J EAE; RREAE.1-RREAE.3) (n = 3) and were generated using Genesis software. (**B**) Mean percent OTU abundance represented as pie chart for each group. (**C**–**G**) One-way ANOVA, followed by Tukey’s multiple comparisons, was performed in order to assess significance in the indicated classes. Bars represent mean ± SEM and exact p-values indicated in text (*p < 0.05; **p < 0.01; ***p < 0.001; ****p < 0.0001).

**Figure 4:**
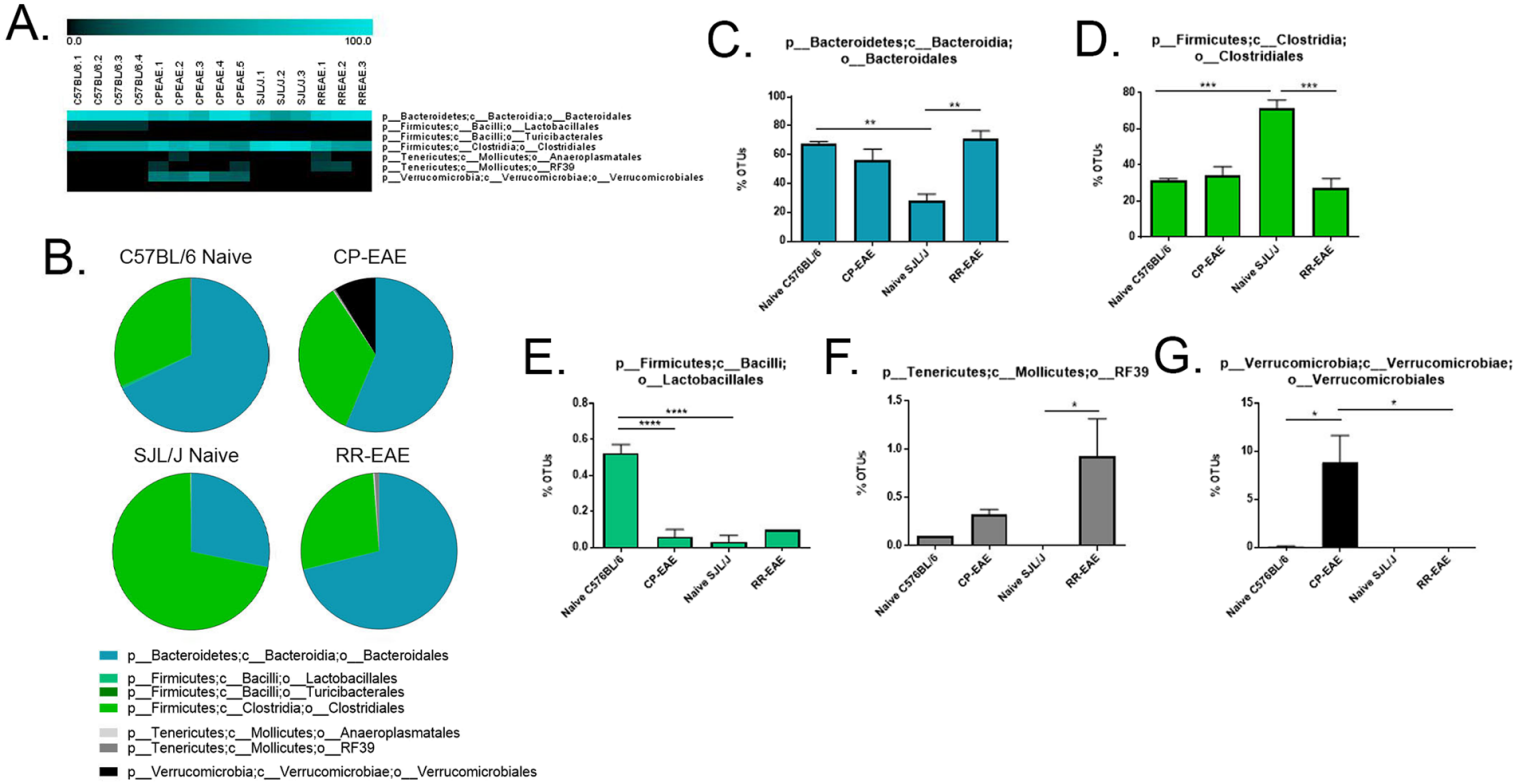
Fecal microbial composition at the Order Level. Stool samples were collected microbial composition determined as described in Fig. 1 legend. (**A**) Heat maps represent percent total Operational Taxonomic Units (OTUs) identified in each sample (Naïve C57BL/6; C57BL/6.1-C57BL/6.4) (n = 4); (C57BL/6 EAE; CPEAE.1-CPEAE.5) (n = 5); (Naïve SJL/J; SJL/J.1-SJL/J.3) (n = 3) and (SJL/J EAE; RREAE.1-RREAE.3) (n = 3) and were generated using Genesis software. (**B**) Mean percent OTU abundance represented as pie chart for each group. (**C**–**G**) One-way ANOVA, followed by Tukey’s multiple comparisons, was performed in order to assess significance in the indicated orders. Bars represent mean ± SEM and exact p-values indicated in text (*p < 0.05; **p < 0.01; ***p < 0.001; ****p < 0.0001).

**Figure 5:**
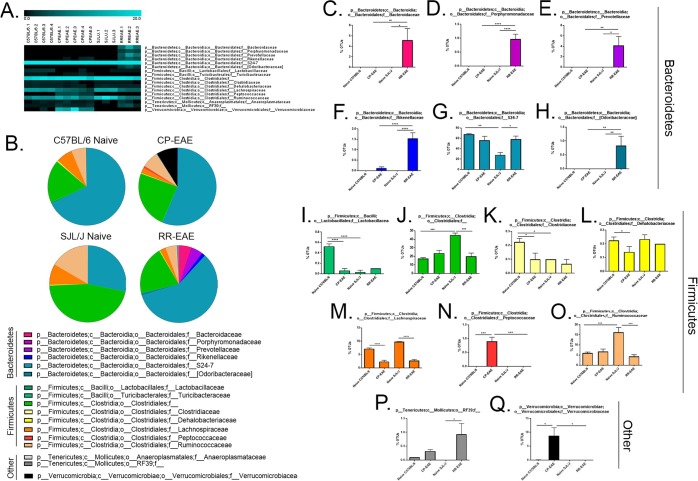
Fecal microbial composition at the Family Level. Stool samples were collected microbial composition determined as described in Fig. 1 legend. (**A**) Heat maps represent percent total Operational Taxonomic Units (OTUs) identified in each sample (Naïve C57BL/6; C57BL/6.1-C57BL/6.4) (n = 4); (C57BL/6 EAE; CPEAE.1-CPEAE.5) (n = 5); (Naïve SJL/J; SJL/J.1-SJL/J.3) (n = 3) and (SJL/J EAE; RREAE.1-RREAE.3) (n = 3) and were generated using Genesis software. (**B**) Mean percent OTU abundance represented as pie chart for each group. (**C**–**Q**) One-way ANOVA, followed by Tukey’s multiple comparisons, were performed in order to assess significance in the indicated families. Bars represent mean ± SEM and exact p-values indicated in text (*p < 0.05; **p < 0.01; ***p < 0.001; ****p < 0.0001).

**Figure 6:**
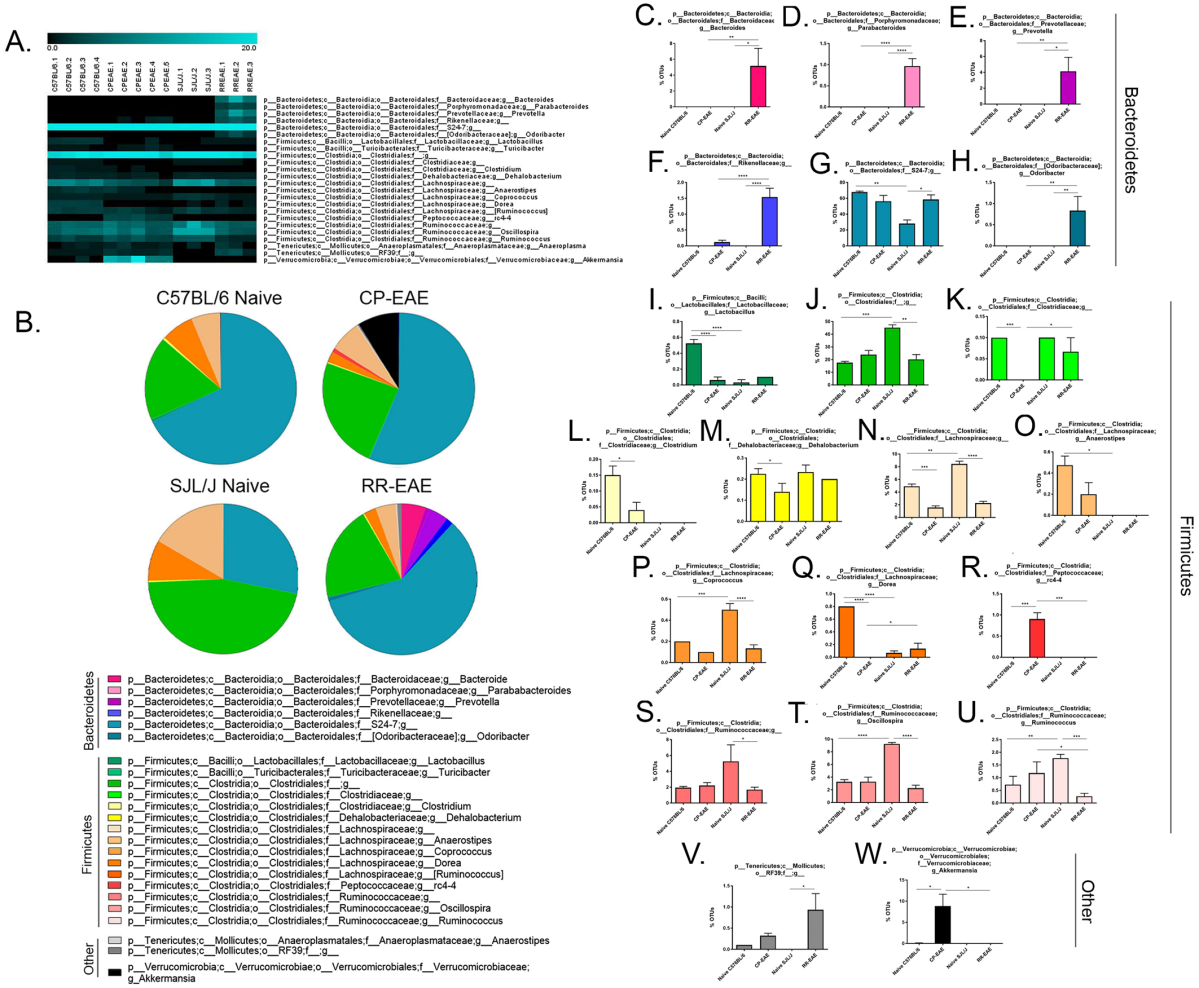
Fecal microbial composition at the Genus Level. Stool samples were collected microbial composition determined as described in Fig. 1 legend. (**A**) Heat maps represent percent total Operational Taxonomic Units (OTUs) identified in each sample (Naïve C57BL/6; C57BL/6.1-C57BL/6.4) (n = 4); (C57BL/6 EAE; CPEAE.1-CPEAE.5) (n = 5); (Naïve SJL/J; SJL/J.1-SJL/J.3) (n = 3) and (SJL/J EAE; RREAE.1-RREAE.3) (n = 3) and were generated using Genesis software. (**B**) Mean percent OTU abundance represented as pie chart for each group. (**C**–**W**) One-way ANOVA, followed by Tukey’s multiple comparisons, was performed in order to assess significance in the indicated genera. Bars represent mean ± SEM and exact p-values indicated in text (*p < 0.05; **p < 0.01; ***p < 0.001; ****p < 0.0001).

**Figure 7:**
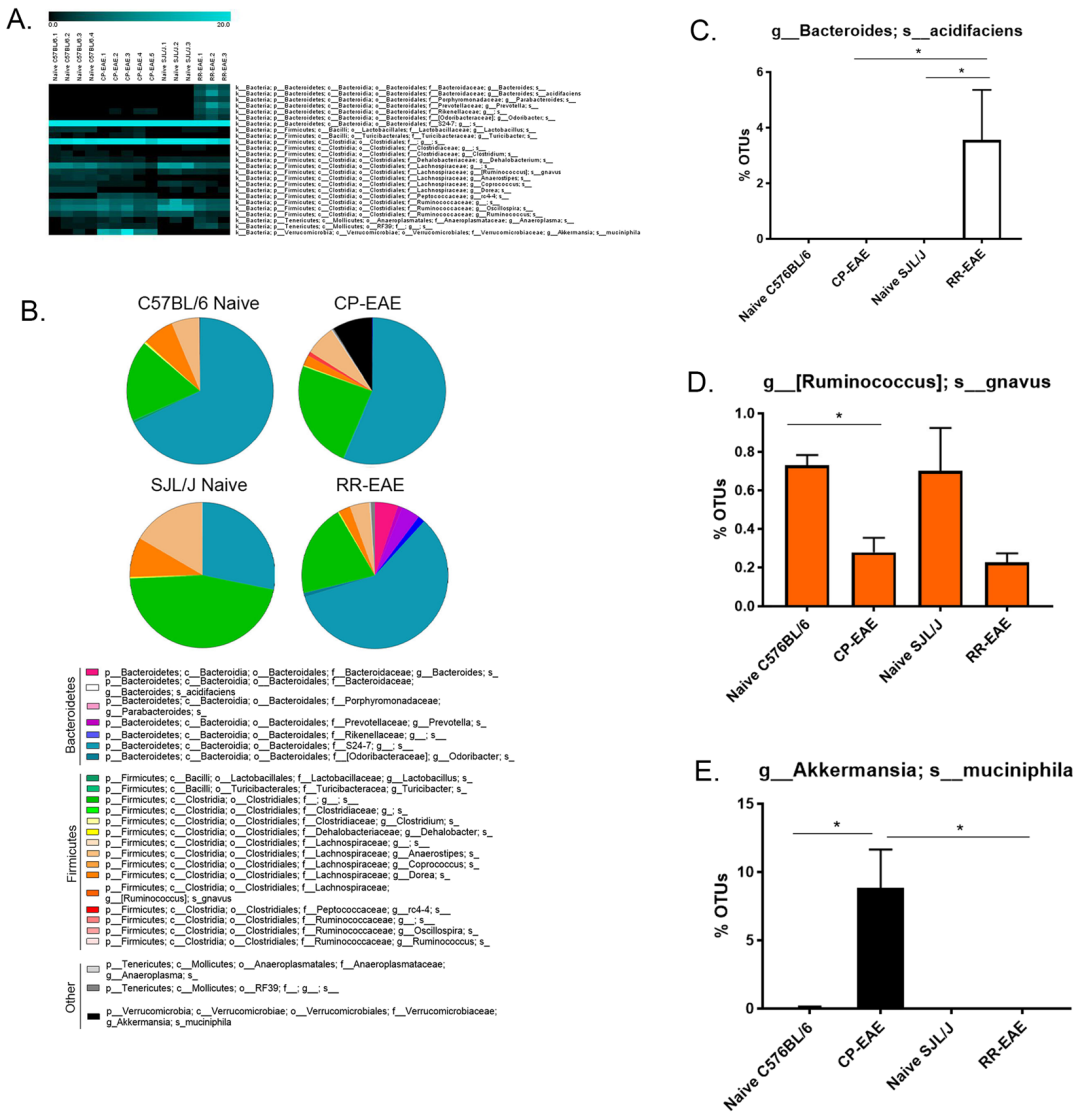
Fecal microbial composition at the Species Level. Stool samples were collected microbial composition determined as described in Fig. 1 legend. (**A**) Heat maps represent percent total Operational Taxonomic Units (OTUs) identified in each sample (Naïve C57BL/6; C57BL/6.1-C57BL/6.4) (n = 4); (C57BL/6 EAE; CPEAE.1-CPEAE.5) (n = 5); (Naïve SJL/J; SJL/J.1-SJL/J.3) (n = 3) and (SJL/J EAE; RREAE.1-RREAE.3) (n = 3) and were generated using Genesis software. (**B**) Mean percent OTU abundance represented as pie chart for each group. (**C**–**E**) One-way ANOVA, followed by Tukey’s multiple comparisons, was performed in order to assess significance in the indicated species. Bars represent mean ± SEM and exact p-values indicated in text (*p < 0.05; **p < 0.01; ***p < 0.001; ****p < 0.0001).

In order to evaluate the microbiome profiles in different clinical courses of EAE, following isolation of prokaryotic 16S rRNA from feces of naïve C57BL/6, naïve SJL/J, CP-EAE (C57BL/6) or RR-EAE (SJL/J), the V4 variable region was sequenced and using Nephele, the microbiome data analysis platform from the National Institutes of Health, data was subjected to taxonomic classification. OTUs for each treatment group were transformed to percent total OTUs and four phyla were represented in our samples (>99.9% of reads) (Fig. 2, Supplemental Table S5). While all reads mapped to organized bacterial classes, 5 were represented in our samples, making up >99.9% of reads. (Fig. 3, Supplemental Table S6). There was a total of 20 orders represented in our groups; however, five orders dominated, representing >99.9% of total OTUs (Fig. 4, Supplemental Table S7). In higher levels of phylogenetic classification, >99% of OTUs correlated to classified bacterial taxa; however, 18%, 24%, 46% and 21% of OTUs from naïve C57BL/6, CP-EAE, naïve SJL/J and RR-EAE, respectively, mapped to unclassified families of bacteria (Fig. 5, Supplemental Table S8). While 17 families of bacteria were represented in our samples, only 13 were significantly differentially detected (Fig. 5C–Q). A large portion of OTUs mapped to unclassified genera in our samples, with 93%, 85%, 88%, and 86% of OTUs from C57BL/6, CP-EAE, naïve SJL/J and RR-EAE, respectively, mapping to unclassified bacterial genera. Twenty-four genera were represented in our samples, with 18 of those being significantly different between groups (Fig. 6, Supplemental Table S9). Species level taxonomic classification revealed 25 species represented in our samples with 20 of those being significantly differentially present (Fig. 7 and S10, Supplemental Table S11). The majority of OTUs mapped to unclassified species, with 22 of 25 species corresponding to unclassified species.

### Members of Phylum *Firmicutes* are Largely Represented in Naïve SJL/J Mice

OTUs corresponding to the *Firmicutes* phylum represented approximately 30% of total OTUs identified in naive C57BL/6 (32% ± 1.3), CP-EAE (34% ± 5.1) and RR-EAE (28% ± 4.4) groups, while the percentage significantly increased in the naïve SJL/J group (72%) (p = 0.0004 vs naïve C57BL/6; p = 0.0003 vs RR-EAE) (Fig. 2B,D, Supplemental Table S5). The ratio of *Firmicutes* to *Bacteroidetes* was 0.5, 0.6 and 0.4 in the naïve C57BL/6, CP-EAE and RR-EAE, respectively, although it was considerably increased in naïve SJL/J mice at 2.6, perhaps contributing to diverging disease phenotypes.

In each group, the percentage of OTUs representing phylum *Firmicutes*, class *Clostridia*, order *Clostridiales* were nearly equal to the percentage of OTUs representing the total *Firmicutes* phylum, indicating that nearly all *Firmicutes* detected were represented by the class *Clostridia* and order *Clostridiales* (Figs 2D, 3E and 4D, Supplemental Tables S5, S6, and S7). OTUs comprising the majority of the order *Clostridiales* mapped to unclassified families and genera and accounted for nearly all family- and genus-level unclassified reads; however, the pattern of representation was similar to previously discussed taxa of phylum *Firmicutes*, with naïve SJL/J (46.1% ± 2.37) mice harboring significantly higher proportions than naïve C57BL/6 (17.83% ± 1.03; p = 0.0003), CP-EAE (23.98% ± 3.34) and RR-EAE (20.3% ± 3.87; p = 0.001) (Figs 5J and 6J, Supplemental Tables S8 and S9).

Albeit at lower abundances, the family *Ruminococcaceae*, also of order *Clostridiales*, exhibited a similar pattern, with naïve SJL/J mice having a significantly higher percentage of OTUs (16.5% ± 2.28) relative to naïve C57BL/6 (6.05% ± 0.55; p = 0.001), CP-EAE (6.68% ± 1.26) and RR-EAE (4.33% ± 0.83; p = 0.0007) mice (Fig. 5O, Supplemental Table S8). Belonging to the *Ruminococcaceae* family are the genera *Oscillospira* and *Ruminococcus* which were both present in moderate proportions and were significantly more abundant in naïve SJL/J mice relative to naïve C57BL/6 (p < 0.0001, *Oscillospira*; p = 0.008, *Ruminococcus*) and RR-EAE (p < 0.0001, *Oscillospira*; p = 0.0008, *Ruminococcus*) mice (Fig. 6T,U, Supplemental Table S9).

While family *Lachnospiraceae*, also belonging to the *Clostridiales* order, was of similar abundance to that of family *Ruminococcaceae*, significant decreases were detected in EAE mice relative to naïve mice in both strains (p < 0.0001, both strains), and it was therefore unlikely to contribute to the differences in disease course (Fig. 5M, Supplemental Table S8). This pattern was also observed at the genus level, with the majority of OTUs corresponding to the *Lachnospiraceae* family partitioning into an unclassified genus or genera and decreasing in both disease models (Fig. 6N, Supplemental Table S9); meanwhile, the same is true for *Clostridiales* order member, genus *Dehalobacterium* of family *Dehalobacteriaceae* (Figs 5L and 6M, Supplemental Tables S8 and S9). Significantly increased proportions of genus *Dorea* of family *Lachnospiraceae* (Fig. 6Q, Supplemental Table S9) were observed in feces from naïve C57BL/6 mice relative to naïve SJL/J and CP-EAE mice, further indicating microbial diversity between naïve mice of these two strains. While both at low abundance, genera *Anaerostipes* and *Coprococcus*, also of family *Lachnospiraceae* belonging to *Firmicutes* were differentially represented in fecal samples in our study. While *Coprococcus* was significantly underrepresented in EAE relative to naïve mice, this was the case in both strains indicating an unlikely role in mediating differing courses of disease (Fig. 6P, Supplemental Table S9). Contrary, genus *Anaerostipes* was present only in mice from the C57BL/6 strain and was decreased in EAE (Fig. 6O, Supplemental Table S9). Additionally, a very small fraction of OTUs representing the *Clostridiaceae* family, also of order *Clostridiales*, correlated to unclassified genera with significantly decreased presence in CP-EAE relative to naive C57BL/6 (p = 0.0004) and RR-EAE (p = 0.02) (Figs 5K and 6K, Supplemental Table S8 and S9). A small proportion of OTUs representing family *Clostridiaceae*, genus *Clostridium* were detected only in mice of the C57BL/6 strain; however, the proportion was significantly decreased upon induction of EAE in this strain (CP-EAE) (Fig. 6L, Supplemental Table S9). Finally, from order *Clostridiales*, genus *rc4–4* of the *Peptococcaceae* family was exclusively represented by the CP-EAE group, suggesting a potential role for this bacterium in driving a chronic-progressive course of disease (Figs 5N and 6R, Supplemental Tables S8 and S9).

In addition to class *Clostridia*, a very small percentage of phylum *Firmicutes* was represented by class *Bacilli* with naïve C57BL/6 mice (0.6% ± 0.04) harboring significantly higher proportions relative to CP-EAE (0.16% ± 0.07, p = 0.0003) and naïve SJL/J (0.03% ± 0.03, p < 0.0001) groups (Fig. 3D, Supplemental Table S6). This observation was mirrored at the order [*Lactobacillales*, naive C57BL/6 mice (0.5% ± 0.07) relative to naïve SJL/J (0.03% ± 0.03, p < 0.0001) and CP-EAE (0.08% ± 0.04) (p < 0.0001)] (Fig. 4E, Supplemental Table S7), and family and genus levels, *Lactobacillaceae* and *Lactobacillus*, respectively [naïve C57BL/6 mice (0.53% ± 0.05) relative to CP-EAE (0.04 ± 0.04; p < 0.0001) and naïve SJL/J mice (0.03% ± 0.03; p < 0.0001)] (Figs 5I and 6I, Supplemental Tables S8 and S9).

Among the significantly altered genera belonging to phylum *Firmicutes*, nearly all partitioned into unclassified species (Supplemental Fig. S10 and Table S11). Unclassified species represented nearly total OTUs belonging phylum *Firmicutes* and genera *Lactobacillus*, *Clostridium*, *Anaerostipes*, *Coprococcus*, *Dorea*, *rc4–4*, *Oscillospira* and *Ruminococcus*, as well as those belonging to order *Clostridiales* and family *Lachnospiraceae* (Supplemental Fig. S10 and Table S11). While not significantly differentially abundant at the genus level, the species *gnavus* belonging to genus *[Ruminococcus]* was significantly enriched in both strains of naïve mice relative to diseased mice, and while unlikely to contribute to differing courses of EAE (Fig. 7B,D, Supplemental Table S11), may be involved in the pathogenesis of EAE, regardless of the clinical course.

Taken together these data indicate differential representation of microbes belonging to the *Firmicutes* phylum in naïve C57BL/6, naïve SJL/J, CP-EAE, and RR-EAE mice, with those belonging to order *Clostridiales* significantly enriched in naïve SJL/J mice, highlighting a role for this particular bacterial order in priming mice for a relapse-remitting EAE disease course.

### Distinct members of Phylum *Bacteroidetes* are Underrepresented in Naïve SJL/J Mice and Exclusively Present in RR-EAE

Contrary to phylum *Firmicutes*, while the percentage of OTUs that mapped to the *Bacteroidetes* phylum were similar in naive C57BL/6 (68% ± 1.4), CP-EAE (56% ± 7.6) and RR-EAE groups (71% ± 4.7), this percentage was significantly decreased in the naïve SJL/J group (28% ± 4.5) (p = 0.004 vs naïve C57BL/6; p = 0.004 vs RR-EAE) (Fig. 2B,C, Supplemental Table S5). The percentage of OTUs representing the phylum *Bacteroidetes* for each group corresponded directly to the percentages of OTUs representing the class *Bacteroidia* and order *Bacteroidales*, designating all *Bacteroidetes* detected to this class and order and significantly less in naïve SJL/J mice (Figs 3B,C, 4B,C, Supplemental Tables S6 and S7).

All 6 families detected in our samples representing phylum *Bacteroidetes*, class *Bacteroidia*, and order *Bacteroidales* were significantly differentially present amongst groups (Fig. 5, Supplemental Table S8). The family accounting for the largest proportion of *Bacteroidales* was family *S24-7* and the percentage of OTUs mirrored the pattern seen at the *Bacteroidetes* phylum level, with significantly less presence in naïve SJL/J mice (28.17% ± 4.53) relative to naïve C57BL/6 (67.68% ± 1.34; p = 0.005), CP-EAE (56.14% ± 7.54) and RR-EAE (58.47% ± 5.82; p = 0.04) (Fig. 5B,G, Supplemental Table S8). A large percentage of OTUs mapped to unclassified genera and species belonging to the *S24-7* family from the *Bacteroidetes* phylum, which also mimicked the trend seen at the phylum level, with significantly lower proportions present in feces from naïve SJL/J mice (Figs 6B,G and S10, Supplemental Table S11).

While detected in lower abundances, the families *Bacteroidaceae*, *Porphyromonadaceae*, *Prevotellaceae* and *[Odoribacteraceae]* were present exclusively in RR-EAE mice with 5.13% (±2.24), 0.97% (±0.18), 4.13% (±1.75) and 0.83% (±0.33), respectively (Fig. 5B–E,H, Supplemental Table S8). Unclassified species of genera *Parabacteroides* of family *Porphyromonadaceae*, *Prevotella* of family *Prevotellaceae*, and *Odoriobacter* of family *Odoribacteraceae* accounted for the total reads at the genus and family levels, and were all exclusively present in RR-EAE mice (Figs 6B,D,E,H and S10, Supplemental Tables S9 and S11). All OTUs corresponding to the family *Bacteroidaceae* mapped to the genus *Bacteroides*, and were therefore also present only in the RR-EAE group (Figs 5C and 6C, Supplemental Tables S8 and S9). A very small fraction of OTUs mapping to the *Bacteroides* genus were represented by an unclassified species (Supplemental Fig. S10 and Table S11), while the larger fraction mapped specifically to species *acidifaciens* (Fig. 7C, Supplemental Table S11). OTUs mapping to both the unclassified species and the *acidifaciens* species (3.56% ± 3.11; p = 0.03 vs naïve SJL/J and p = 0.02 vs CP-EAE) were present only in feces from RR-EAE mice. Furthermore, while the family *Rickenellaceae* was detectable in feces from CP-EAE mice (0.12% ± 0.06), it was significantly enriched in RR-EAE mice (1.53% 0.28; p < 0.0001) (Fig. 5F, Supplemental Table S8) and OTUs mapping to unclassified genera and species represented the entirety of family *Rickenellaceae*, and were therefore also significantly more abundant in the RR-EAE group (Figs 6F and S10, Supplemental Tables S9 and S11). While these changes are visible in the bar graphs, the pie charts in Figs 5B and 6B provide a more robust depiction of the expansion of the population of *Bacteroidetes* family members in the RR-EAE group, suggesting these bacteria may be responsible for driving the primary relapse seen in the PLP-immunized SJL/J strain.

Taken together these data showed higher taxa-level differences in microbial distribution with the S24-7 family of phylum *Bacteroidetes* significantly underrepresented in the naïve SJL/J mice and the expansion of all families belonging to the *Bacteroidales* order in RR-EAE, highlighting a potential role for this particular bacterial order in initiating a relapse-remitting EAE disease course.

### *Akkermansia muciniphila* is Exclusively Present in the Chronic-Progressive form of EAE

Interestingly, and in line with other reports, the phylum *Verrucomicrobia* was represented exclusively in the CP-EAE group, comprising nearly 10% of the total (8.8% ± 2.8) OTUs (p = 0.03 vs naïve C57BL/6; p = 0.04 vs RR-EAE) (Fig. 2B,F, Supplemental Table S5)^19^. The *Verrucomicrobia* phylum was represented entirely by class *Verrucomicrobiae* (Fig. 3B,G, Supplemental Table S6) order *Verrucomicrobiales* (Fig. 4B,G, Supplemental Table S7), family *Verrucomicrobiaceae* (Fig. 5B,Q, Supplemental Table S8), genus *Akkermansia* (Fig. 6B,W, Supplemental Table S9), and species *muciniphila* (Fig. 7B,E, Supplemental Table S11), indicating a potential significant role for this bacterium in the chronic-progressive mouse model of MS and in shaping a chronic inflammatory environment in EAE.

### Members of *Tenericute*s Phylum are Enriched in the Relapsing-Remitting Form of EAE

Lastly, the phylum *Tenericutes* was observed at low levels in all four groups (<1.5%); however, it was significantly increased in the RR-EAE group when compared to naïve SJL/J (p < 0.05), and increased in CP-EAE relative to naïve C57BL/6 mice (Fig. 2B,E, Supplemental Table S5). Furthermore, the percentage of OTUs representing class *Mollicutes* in phylum *Tenericutes* directly mirrored the percentages observed at the phylum level (Fig. 3B,F, Supplemental Table S6). Orders representing the phylum *Tenericutes* class *Mollicutes* dropped slightly in percentage; however, order *RF39* remained significantly increased in the RR-EAE group with 1% (±0.4) OTUs aligning (p = 0.01 vs naïve SJL/J) (Fig. 4B,F, Supplemental Table S7). An unclassified family (or families) belonging to the order *RF39* represented the entirety of phylum *Tenericutes* and was observed in significantly higher percentage in the RR-EAE mice (0.9% ± 0.38) relative to naïve SJL/J mice (p = 0.01), where it was not detected; and while it did represent a small portion of OTUs in the naïve C57BL/6 (0.1% ± 0.0) and CP-EAE (0.32% ± 0.06) mice, it was not significantly lower relative to RR-EAE (Fig. 5B,P, Supplemental Table S8). Genus level taxonomic categorization identified yet-to-be-classified genera belonging to the *Tenericutes* phylum (Fig. 6B,V, Supplemental Table S9); similarly, all species belonging to phylum *Tenericutes* were unclassified and represented the order *RF39* in total and were significantly more abundant in the RR-EAE group relative to naïve SJL/J (p = 0.01) (Supplemental Fig. S10 and Table S11)

### Biomarker Analysis

**Figure 8:**
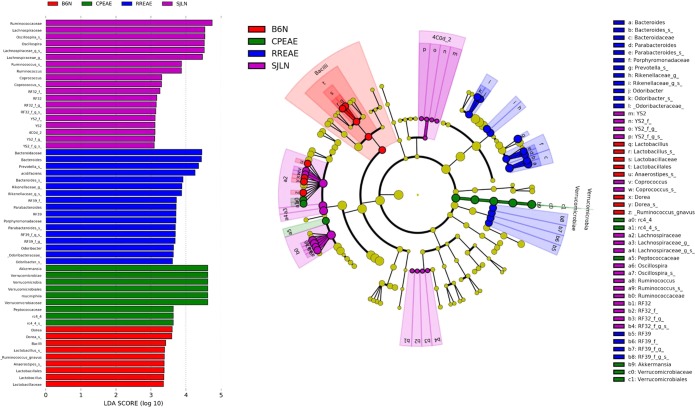
LEfSe Analysis: biomarkers associated with naïve C57BL/6, naïve SJL/J, CP-EAE and RR-EAE (**A**) A linear discriminant effect size (LEfSe) analysis was performed (alpha value ≥ 0.05, logarithmic LDA score threshold ≥ 3.0) on all four groups together (**B**) The cladogram represents the phylogenetic relationship of significant OTUs associated with each group.

In order to identify differentially abundant bacterial taxa from feces that may serve as biomarkers, we used Linear discriminant analysis (LDA) coupled with effect size measurement (LEfSe) to analyze microbial contents between all groups, as well as between select groups^22^. When microbial contents of all four groups (naïve C57BL/6, CP-EAE, naïve SJL/J, and RR-EAE) were analyzed together, 54 total discriminative features were identified with an LDA ≥ 3. Those bacterial taxa that were discriminative for naïve C57BL/6 mice were all members of the *Firmicutes* phylum and included an unclassified species of *Lactobacillus* (class *Bacilli*, order *Lactobacillales*, family *Lactobacillaceae*), as well as unclassified species from genera *Anaerostipes* and *Dorea*, in addition to *Ruminococcus gnavus*, all from the *Lachnospiraceae* family (class *Clostridia*, order *Clostridiales*) (Fig. 8 and Supplemental Table S12). There were 3 bacterial taxa that were consistently increased in abundance in the CP-EAE group, with the highest LDA score belonging to *Akkermansia muciniphila* (phylum *Verrucomicrobia*, class *Verrucomicrobia*, order *Verrucomicrobiales*, family *Verrucomicrobiaceae*) (Fig. 8 and Supplemental Table S12). Members of the *Firmicutes* phylum, *Clostridia* class and *Clostridiales* order, the *Peptococcaceae* family and unclassified species of *rc4_4*, of the *Lachnospiraceae* family, were also identified as biomarkers of the chronic progressive disease course (CP-EAE) (Fig. 8 and Supplemental Table S12). The majority of the bacterial taxa that were identified as consistently more abundant in naïve SJL/J mice were made up of the *Lachnospiraceae* and *Ruminicoccaceae* families (phylum *Firmicutes*, class *Clostridia*, order *Clostridiales*), including unclassified genera and an unclassified species of *Coprococcus* from *Lachnospiraceae* and unclassified species of *Oscillospira* and *Ruminococcus* from *Ruminococcaceae* (Fig. 8 and Supplemental Table S12). The *RF32* and *YS2* classes of the *Proteobacteria* and *Cyanobacteria* phyla, respectively, were also identified as potential microbial biomarkers of naïve SJL/J strain, albeit they were present in very low abundances (<0.1%) (Fig. 8 and Supplemental Table S12). In contrast to naïve SJL/J mice, the majority of the bacterial taxa that partitioned into the RR-EAE group upon LEfSe analysis were comprised primarily of bacteria belonging to the *Bacteroidales* order, including unclassified species from genera *Odoribacter*, *Parabacteroides*, *Prevotella*, and *Bacteroides* belonging to families *Odoribcteraceae*, *Porphyromonadaceae*, *Prevotellaceae*, and *Bacteroidaceae*, respectively (Fig. 8 and Supplemental Table S12). Additionally, unclassified genera from the *Rikencellaceae* family, as well as *Bacteroides acidifaciens* of the *Bacteroidaceae* family, also both of order *Bacteroidales*, were identified as RR-EAE-specific biomarkers using LEfSe analysis (Fig. 8 and Supplemental Table S12). Unclassified members of bacterial family *RF39*, of phylum *Tenericutes* and class *Mollicutes*, were also found to be consistently abundant in the RR-EAE group (Fig. 8 and Supplemental Table S12).

When LEfSe analysis was performed on specific group pairs (naïve C57BL/6 vs naïve SJL/J; naïve C57BL/6 vs CP-EAE; naïve SJL/J vs RR-EAE; and CP-EAE vs RR-EAE) many of the bacterial taxa that were deemed discriminative for a particular group, were the same taxa identified when performing LEfSe analysis on all four groups together, indicating these taxa are robust biomarkers. Furthermore, there were bacterial taxa identified in specific pair-wise comparisons there were not detected in LEfSe analysis of all four groups together. When LEfSe analysis was performed on microbiome of naïve C57BL/6 and naïve SJL/J groups, in addition to those bacterial taxa identified upon analysis of all groups together, 5 bacterial taxa were consistently abundantly represented in naïve C57BL/6 mice, while one was represented in naïve SJL/J mice (Supplemental Fig. S13 and Table S12). With an LDA score greater than 5, the *S24_7* family of phylum *Bacteroidetes*, class *Bacteroides*, and order *Bacteroidales* was identified as biomarker of naïve C57BL/6 mice, when compared to naïve SJL/J mice (Supplemental Fig. S13 and Table S12). Additionally, the *Mogibacteriaceae* (although at a very low abundance <0.1%) and *Peptococcaceae* families, both of class *Clostridia* and order *Clostridiales*, were detected as consistently more abundant in naïve C57BL/6 mice as in naïve SJL/J mice (Supplemental Fig. S13 and Table S12). Also, of class *Clostridia* and order *Clostridiales*, unclassified species of genus *rc4_4* of family *Lachnospiraceae* was discriminative for naïve C57BL/6 mice relative to naïve SJL/J mice (Supplemental Fig. S13 and Table S12). Lastly, the genus *Allobaculum* of phylum *Firmicutes*, class *Erysipelotrichi*, order *Erysipelotrichales*, and family *Erysipelotrichaceae* was also identified as a biomarker of naïve C57BL/6 microbiome; however, these taxa were present at very low abundances (<0.1%), making their physiological relevance unknown (Supplemental Fig. S13 and Table S12). While there were numerous bacterial taxa identified as discriminative for naïve C57BL/6, when compared to naïve SJL/J, there was only one additional bacterial taxon, outside of those identified in the all-group LEfSe analysis, that partitioned into the naïve SJL/J group, and that was an unclassified species of genus *Anaerotruncus* of family *Ruminococcaceae*, order *Clostridiales*, class *Clostridia*, and phylum *Firmicutes* (Supplemental Fig. S13 and Table S12).

When characterizing the difference in bacterial taxa of the microbiome in naïve C57BL/6 mice and CP-EAE mice, outside of those biomarkers identified upon LEfSe analysis of all four groups together (Fig. 8 and Supplemental Table S12), four further taxa were found to differentiate between the two groups. Naïve C57BL/6 mice were found to consistently harbor a significantly higher abundance of an unclassified species of the *Adlercreutizia* genus belonging to phylum *Actinobacteria*, class *Coriobacteriia*, order *Coriobacteriales*, and family *Coriobacteriaceae*, as well as an unclassified species of genus *Coprococcus* (phylum *Firmicutes*, class *Clostridia*, order *Clostridiales*, and family *Lachnospiraceae*) (Supplemental Fig. S14 and Table S12). On the other hand, the *RF39* order of bacteria belonging to the *Tenericutes* phylum and *Mollicutes* class, as well as the *Coprobacillus* genus of phylum *Firmicutes*, class *Erysipelotrichi*, order *Erysipelotrichales*, and family *Erysipelotrichaceae* identified as biomarkers of CP-EAE, when compared to naïve C57BL/6 (Supplemental Fig. S14 and Table S12).

In order to identify bacterial taxa characteristic of normal or disease state in SJL/J mice, LEfSe analysis was performed on bacterial abundances present in feces of naïve SJL/J and RR-EAE mice. Interestingly, there were no additional bacterial taxa, outside of those identified in the all-group LEfSe analysis (Fig. 8 and Supplemental Table S12), detected as characteristic of naïve SJL/J mice from this pair-wise analysis, and only one additional taxon that was discriminative for RR-EAE. The *S24_7* family of phylum *Bacteroidetes*, class *Bacteroidia*, and order *Bacteroidales* was found to be a microbiome biomarker for RR-EAE when compared with naïve SJL/J mice, with an LDA score >5 (Supplemental Fig. S15 and Table S12).

Biomarker and effect size analysis of bacterial abundance in the feces of CP-EAE and RR-EAE mice revealed 5 discriminative features that were not detected in the initial all-group analysis (Fig. 8 and Supplemental Table S12). LEfse analysis detected three further bacterial taxa that were consistently more abundant in mice exhibiting the chronic-progressive form of EAE, relative to mice exhibiting the relapse-remitting form (Supplemental Fig. S16 and Table S12). All of phylum *Firmicutes*, genus *Turicibacter* of class *Bacilli*, and genera *Clostridium* and *Ruminococcus* both of class *Clostridia*, were identified as biomarkers of CP-EAE; furthermore, from phylum *Proteobacteria*, the genus *Eneterbacteriaceae*, as well as genus *Dorea*, from phylum *Firmicutes* (class *Clostridia*, order *Clostridiales*, family *Lachnospiraceae*) were identified as consistently more abundant in feces from diseased SJL/J mice (RR-EAE) when compared to diseased C57BL/6 mice (CP-EAE) (Supplemental Fig. S16 and Table S12). In order to identify the bacterial taxa that are most highly representative of a particular group, regardless of the type of comparison, we have chosen to highlight those taxa present in that group, across all LEfSe analyses performed. In doing so, an unclassified species of *Lactobacillus* (phylum *Firmicutes*, class *Bacilli*, order *Lactobacillales*, family *Lactobacillaceae*), as well as an unclassified species of *Dorea* (phylum *Firmicutes*, class *Clostridia*, order *Clostridiales*, family *Lachnospiraceae*) were identified as robust biomarkers of naïve C57BL/6 mice (Supplemental Table S12, red text). Furthermore, unclassified members of the *Lachnospiraceae* family, an unclassified species of *Oscillospira* (phylum *Firmicutes*, class *Clostridia*, order *Clostridiales*, family *Ruminococcaceae*), and unclassified members of the *RF32* order (phylum *Proteobacteria*, class *Alphaproteobacteria*) were identified as biomarkers of naïve SJL/J mice in all LEfSe analyses; however, the physiological significance of the *RF32* identified biomarker is unclear, given its very low abundance (<0.1%) (Supplemental Table S12, red text). Both from phylum *Firmicutes*, class *Clostridia*, and order *Clostridiales*, the *Peptococcaceae* family, as well as an unclassified species of *Lachnospiraceae* family, *rc4-4*, were identified as biomarkers of CP-EAE across all LEfSe analyses (Supplemental Table S12, red text). Additionally, and most significantly discriminative for CP-EAE was *Akkermansia muciniphila* of phylum *Verrucomicrobia*, class *Verrucomicrobiae*, order *Verrucomicrobiales*, and family *Verrucomicrobiaceae* (Supplemental Table S12, red text). Finally, discriminative for RR-EAE across all LEfSe analyses were an unclassified species of *Prevotella* from family *Prevotellaceae* and *Bacteroides acidifaciens* from family *Bacteroidaceae*, both of which belong to phylum *Bacteroidetes*, class *Bacteroidia*, and order *Bacteroidales* (Supplemental Table S12, red text). LEfSe analysis identified numerous bacterial taxa as biologically relevant biomarkers of naïve C57BL/6, naïve SJL/J, CP-EAE and RR-EAE mice, some of which were identified only in select group comparisons. Interestingly, however, many of the identified biomarkers of a given group were detected across all comparisons, indicating these taxa are likely more robust in their biomarker abilities.

## Discussion

Experimental autoimmune encephalomyelitis (EAE) is a well-characterized mouse model of the neuro-inflammatory demyelinating disease, multiple sclerosis (MS) and is mediated by pro-inflammatory Th1 and Th17 CD4+ T-cells^1^. Patients with MS exhibit different types of clinical disease. The relapse-remitting form (RR-MS) is the most common type in which patients have temporary periods of relapses or exacerbations. In contrast, about 10% of MS patents exhibit chronic and slowly worsening symptoms with no relapses or remissions, called chronic-progressive disease (CP-MS)^2^. Similar to these two forms in humans, mouse models in different strains exhibit diverse disease courses, with the C57BL/6 strain exhibiting a chronic-progressive course and the SJL/J strain exhibiting a relapse remitting course^4^. While the precise mechanisms contributing to the different disease courses are currently unknown, there is evidence indicating a major role for differential balance between pro-inflammatory Th1/Th17 cells and anti-inflammatory Treg cells, with RR-EAE fluctuating between the two T cell phenotypes throughout the course of disease and CP-EAE unable to mount Treg-mediated inflammatory resolution^5,23–26^. Studies from our lab and others have strongly supported the Th1/Th17 CD4+ T cell phenotype in driving EAE, as well as the Treg phenotype in abrogating clinical signs of disease^27–30^. Genetic knockout of CD44 leads to a shift in T cell phenotype from Th1/Th17 to Th2/Treg and protects from EAE^27^. Moreover, our lab has identified various natural compounds that protect from EAE and other autoimmune diseases via microRNA-mediated shifts toward Treg differentiation^30^. While the clinical course of EAE may correlate with regulatory or effector T cell induction, what triggers such differential induction of T cells remains unclear.

Currently, there is a focus on environmental factors affecting CD4+ T cell differentiation and gut microbial composition has gained much attention for its ability to shape T cell phenotype and therefore overall immune responses. In many autoimmune diseases, as well as specifically in EAE and MS, studies have shown that the gut microbiota can drive T cells toward a pro-inflammatory phenotype (Th1/Th17) or anti-inflammatory or tolerant phenotype (Th2/Treg) altering disease parameters, depending on the composition^12–14^. With direct correlation between clinical course of EAE or MS and Treg cells^21,26^, we hypothesized that diverse microbial communities differentially shape the immune response in EAE and this may play a role in determining a chronic-progressive or relapse-remitting course of disease. Interestingly, in this study, we find significantly diverse microbial populations in the two strains of mice before and after the induction of CP and RR forms of EAE.

In the current study we used sequencing of the V4 variable region of prokaryotic 16S RNA from feces of naïve C57BL/6, naïve SJL/J, CP-EAE and RR-EAE mice using the Illumina MiSeq and analysis of sequencing results using NIH-supported microbiome data analysis software, Nephele, to study the microbiota. Both disease courses exhibited an initial peak at day 13 post-immunization; however, the CP-EAE mice continued to slowly progress while the RR-EAE mice exhibited several remittances and relapses, indicated by an increase or decrease in clinical scores >1 (Supplemental Fig. S1)^3^. While scores at the initial peaks of disease were the same, we found that T regulatory (T reg) master transcription factor and marker, FoxP3, was significantly increased in encephalitogenic CD4+ T cells in the RR form of the disease relative to the CP form (Supplemental Fig. S2). T reg cells are known to inversely correlate with MS disease state, with increased levels during periods of remission and decreased levels during periods of relapse^21^; in fact, the first remittance takes place immediately following the time point at which feces was collected and FoxP3 levels significantly increased in RR encephalitogenic CD4+ cells (Supplemental Fig. S1). Therefore, because the two models have identical presentation up until the first relapse in RR-EAE, sampling microbiota immediately prior to the divergence in disease courses allows for the identification of bacterial taxa present in RR-EAE which may shape a tolerant immune state through Treg generation and promote remission, as well as taxa present in CP-EAE which may drive a more pro-inflammatory state and promote progression of disease via generation of non-regulatory T cells (i.e. Th1 and Th17).

We found significant differences in *Chao1* and Observed OTUs, both metrics of α-diversity (Fig. 1A,B). Furthermore, significant differences in β-diversity were identified using PERMANOVA analysis of unweighted UniFrac distance metrics (Fig. 1E). Additionally, statistical analysis of UniFrac distances indicated that samples within each group were significantly more similar than samples between groups, ruling out any inter-individual variability effect (Fig. 1F,H). Importantly, we found significant differentially abundant proportions of bacteria belonging to the *Firmicutes* and *Bacteroidetes* phyla in naïve SJL/J and C57BL/6 strains of mice (Fig. 2C,D). Naïve SJL/J mice harbored significantly more bacteria classified to the *Firmicutes* phylum relative to naïve C57BL/6 mice. These bacteria all mapped to the class *Clostridia* and order *Clostridiales*, with approximately 72% (±4.47) of OTUs detected in feces of naïve SJL/J mice corresponding to these taxa, relative to 31% (±1.27) in naïve C57BL/6 mice (Figs 3E and 4D). Some families and genera belonging to these taxa which were present in significantly higher amounts in naïve SJL/J feces included the family *Lachnospiraceae* and unclassified genera, as well as the genus *Coprococcus* (Figs 5M and 6N,P). Moreover, the family *Ruminococcaceae* and genera *Oscillospira* and *Ruminococcus* were significantly enriched in naïve SJL/J mice relative to naïve C57BL/6 mice (Figs 5O, 6T,U). In addition to these observable differences in bacterial taxa, an algorithm designed for biomarker discovery (LEfSe) identified these same taxa as characterizing naïve SJL/J mice (Supplemental Fig. 13 and Table S12), indicating that not only is there an observable difference in the abundance of these taxa, but that they are, in fact predicted biomarkers of naïve SJL/J mice relative to naïve C57BL/6 mice and may have the ability to serve as indicators of the type of disease course to which an individual is predisposed. LEfSe analysis also identified an order of *Proteobacteria*, *RF32*, as a discriminative feature for naïve SJL/J mice when compared to all other groups. While this taxon may be discriminative based on LEfSe analysis, this taxon was not included in the initial descriptive analysis, as only taxa corresponding to OTUs representing ≥ 0.1% of total OTUs were evaluated for significance, as bacteria representing such a low proportion of the microbiome is not likely physiologically relevant. Studies in mice have indicated that *Firmicutes* organisms, specifically those of family *Clostridiales*, shape Treg generation in the gut and mice colonized with these organisms develop less severe DSS-induced colitis^31^. Therefore, the increased presence of these organisms in naïve SJL/J mice may contribute to the enhanced ability of the SJL/J strain to mount a Treg response after disease initiation, leading to periods of remittance. Additionally, the presence of *Firmicutes* in naïve SJL/J mice may influence the outgrowth of certain types of bacteria during disease, perhaps priming this strain of mouse for a relapse-remitting course of EAE. Moreover, *Firmicutes* are butyrate producers and therefore may function to maintain gut integrity in naïve SJL/J mice^32^. Further evaluation of short-chain fatty acids present in the serum or feces of our mice may lead to a better understanding of their role.

Conversely, naïve mice of the C57BL/6 strain harbored significantly higher proportions of bacteria belonging to the *Bacteroidetes* phylum, all of which were represented by class *Bacteroidia* order *Bacteroidales*. Around 68% (±1.37) of OTUs detected in the feces of naïve C57BL/6 mice corresponded to the aforementioned taxa, while only 28% (±4.53) of OTUs present in naïve SJL/J mice represented these taxa (Figs 2C, 3C and 4C). The bulk of organisms belonging to order *Bacteriodales* were represented by the *S24-7* family and unclassified genera (Fig. 5G). The *S24-7* family was also predicted by LEfSe analysis to be a biomarker of naïve C57BL/6 mice when compared to naïve SJL/J mice, with a > 5 LDA score (Supplemental Fig. 13 and Table S12). While not much is known about the *S24-7* family of bacteria, it was shown to be increased in high-fat diet fed mice that became diabetic, indicating a potential role for this bacteria is shaping a pro-inflammatory phenotype^33^, perhaps priming C57BL/6 mice for a chronic-progressive EAE disease course. While the homeostatic functions of bacteria belonging to the *Clostridiales* and *Bacteroidales* order are not well characterized, it is important to note a significant difference in proportion of these organisms in naïve C57BL/6 and SJL/J mice, as they may play a role in shaping differential immune responses in EAE. Additional studies such as fecal transfer experiments or alterations in the gut microbial composition, in the two strains of mice, is necessary to gain a more precise understanding of the role of gut microbiota in shaping EAE disease course.

Just as there were differentially present abundances of bacteria in naïve mice SJL/J and C57BL/6 strains, there were also interesting differences in microbial composition in diseased CP-EAE and RR-EAE mice. While there was a very small proportion observed in naïve C57BL/6 mice (0.07%), the phylum *Verrucomicrobia* was significantly represented in feces from CP-EAE mice and absent from naïve SJL/J and RR-EAE fecal samples (Fig. 2F). Specifically, *Akkermansia muciniphila* of phylum *Verrucomicrobia* was significantly present in CP-EAE at each taxonomic level, with 8.8% (±2.84) of OTUs representing this bacterium (Figs 3G, 4G, 5Q, 6W and 7E); furthermore, it was identified as a biomarker of CP-EAE across all LEfSe analyses (Fig. 8, Supplemental Figs S14 and S16 and Table S12), strongly indicating that *A*. *muciniphila* is a robust biomarker of the chronic-progressive course of EAE. *Akkermansia muciniphila* is a mucin-degrading bacterium and is also found to be elevated in humans with MS^19,34,35^. The presence of *Akkermansia muciniphila* in CP-EAE is significant, as it has been shown to alter gut permeability via degradation of the mucus layer, allowing microbial antigens to interact with the intestinal immune system^34^. In studies using *Salmonella*-induced gut inflammation, the presence of *A*.*muciniphila* greatly exacerbated parameters of disease and this was dependent on *A*. *muciniphila*-mediated disturbance of mucus homeostasis^36^. The presence of *A*. *muciniphila* in naïve C57BL/6 mice and absence in naïve SJL/J mice may play a major role in shaping the immune response in EAE. In the disease state, *A*. *muciniphila* exhibited an outgrowth in CP-EAE mice, perhaps leading to the pathogenicity of normally commensal microorganisms. It will be interesting to perform fecal transfers or *A*. *muciniphila* colonization in CP-EAE and RR-EAE mice in order to evaluate the effect on EAE disease course. Any or all of the numerous bacteria present in CP-EAE mice could shape a pro-inflammatory immune state in the absence of appropriate gut mucus homeostasis, as gut mucosal barrier plays an integral role in preventing inflammation in response to gut commensals^37^. One potential pathogenic candidate identified in CP-EAE mice is the genus *rc4-4* from phylum *Firmicutes* and family *Peptococcaceae*. *rc4-4* is present only in feces from CP-EAE mice (0.9% ± 0.15) and absent from RR-EAE feces (Fig. 6R). Interestingly, *rc4-4* was identified as a biomarker of CP-EAE across all LEfSe analyses (Fig. 8, Supplemental Figs S14 and S16 and Table S12), bolstering its potential as a *bona fide* biomarker of the chronic-progressive form of EAE. Although present at a low abundance in our samples, *rc4-4* was also identified in feces from mice experiencing indomethacin-induced gut inflammation, suggesting a potential pro-inflammatory role for this genus of bacteria^38^. While many studies have indicated an inverse relationship between *A*. *muciniphila* and inflammation, these are mostly in the context of metabolic diseases where *A*. *muciniphila* restores glucose tolerance^39,40^. While further experiments are necessary, taken together, these data indicate a potential role for the gut commensal *A*. *muciniphila* in driving a chronic-progressive course of EAE.

While *A*. *muciniphila* was significantly present in CP-EAE, there was an expansion of family members belonging to the *Bacteroidales* order of bacteria specifically in RR-EAE mice (Fig. 4B). Genera of order *Bacteroidales* significantly increased in RR-EAE relative to CP-EAE include *Bacteroides*, *Parabacteroides*, *Prevotella*, *Rikenellaceae* and *Odoribacter* (Figs 6C-F and 6H). In addition to the observable differential abundances of the aforementioned bacterial taxa in RR-EAE, LEfSe analysis also identified them as both statistically significant and biologically relevant, characterizing them as discriminative for RR-EAE when compared to CP-EAE (Supplemental Fig. S16 and Table S12), suggesting they have potential to be biomarkers of a relapse-remitting disease course. Many of these genera have been linked to pro-inflammatory processes and are increased in models of colitis and high-fat diet-induced obesity and because RR-EAE is an inflammatory disease, the presence of these bacteria is not surprising^41–43^. On the other hand, some of these bacteria have also been found to inversely correlate with inflammatory liver disease^44^ and to be associated with protection from EAE. Specifically, many species of the genus *Bacteroides* have been found to be elevated in EAE-resistant CD44KO mice^20^, including *Bacteroides acidifaciens*, which we also found to be significantly more abundant in RR-EAE (3.56% ± 3.11) relative to all other groups, where it was undetectable (Fig. 7C). LEfSe analyses found *B*. *acidifaciens* to be a biomarker of RR-EAE across all analyses (Fig. 8, Supplemental Figs S15 and S16 and Table S12), implicating it as a strong biomarker of the relapse-remitting course of EAE. Furthermore, *Bacteroides acidifaciens* has been implicated in maintaining immune homeostasis, as there was a decrease in abundance during the progression of DSS-induced colitis, indicating a beneficial role for gut commensal *B*. *acidifaciens*^45^. Additionally, perhaps the best characterized EAE-protective commensal, *Bacteroides fragilis*, belongs to the same order of bacteria which was significantly expanded in feces of mice displaying the RR-EAE disease course. EAE mice exposed to prophylactic or therapeutic treatment with *B*. *fragilis* generated CD103+ dendritic cells, which convert naïve CD4+ T cells into potent Foxp3+ Treg cells, protecting from disease^12,14^. Furthermore, there is evidence that *Bacteroidetes* organisms possess butyrogenic potential through the use of several non-canonical butyrate-producing pathways, including those that use amino acids as substrates, as amino acids result from *Bacteroidetes’* primary fermentation^32^. While further experiments are vital in determining a direct role for *Bacteroidales* family members in shaping T cell differentiation and mediating RR-EAE, taken together, these data suggest a potential role for this family in shaping the immune response that drives a relapse-remitting disease course.

Notably, we also identified significant alterations in gut microbiota following immunization to induce EAE, some of which were strain-specific and others that were independent of strain. For example, several family members belonging to order *Clostridiales*, particularly those of the *Lachnospiraceae* family were decreased in EAE compared to naïve and this change was present in both C57BL/6 and SJL/J strains, reinforcing microbial dysbiosis as a potential critical environmental component of EAE disease pathogenesis^15–19^. This observation is also in-line with bacterial taxa identified by LEfSe analysis, whereby 10 discriminative events were represented by the *Clostridiales* order and 8 events by the *Lachnospiraceae* family in naïve C57BL/6 mice, while only 3 *Clostridiales* and 2 *Lachnospiraceae* discriminative events were detected in CP-EAE mice (Supplemental Fig. S14 and Table S12). Furthermore, 11 discriminative events were represented by order *Clostridiales* and 3 events by family *Lachnospiraceae* in naïve SJL/J mice, there were no events represented by these taxa characterized in RR-EAE, indicating a loss of *Clostridiales*, and more specifically *Lachnospiraceae*, in the disease state relative to the naïve state (Supplemental Fig. S15 and Table S12), regardless of strain. Specific to the SJL/J strain of mouse, induction of EAE led to a shift in the ratio of *Firmicutes: Bacteroidetes*, represented by increases in organisms of the *Bacteroidales* order and decreases in unclassified *Clostridiales* members and *Ruminococcaceae* family members. In fact, when pair-wise LEfSe analysis of naïve SJL/J and RR-EAE was performed, all but one bacterial taxon identified as a biomarker of RR-EAE belongs to order *Bacteroidales*, while a large proportion of biomarkers identified in naïve SJL/J are members of *Clostridiales* and *Ruminococcaceae* (Supplemental Fig. S15 and Table S12). Besides a significantly increased abundance of *A*. *muciniphila*, the bulk of OTUs in the C57BL/6 strain remained relatively unchanged following induction of EAE, with significantly lower levels of bacteria belonging to the *Lactobacillus* genus, as well as those belonging to the genera *Anaerostipes* and *Dorea* from the *Lachnospiraceae* family. Not surprisingly, LEfSe analysis identified both *Dorea* and *Lactobacillus* genera as biomarkers of naïve C57BL/6 mice (Fig. 8, Supplemental Figs S13 and S14 & Table S12). Changes in gut microbial composition that are shared between strains, as well as those that are strain-specific further support a role for microbial dysbiosis in the pathogenesis of EAE^15–19^. In conclusion, our study identifies significantly different gut microbial populations in naïve C57BL/6 and naïve SJL/J mice, which may shape the immune response to EAE induction. Naïve C57BL/6 mice harbored significantly more *Bacteroidetes* and significantly less *Firmicutes* organisms than did naïve SJL/J mice. Additionally, significant differences in gut microbial populations were identified in the chronic-progressive form of EAE when compared to the relapse-remitting form of EAE. *A*. *muciniphila* was significantly more abundant in CP-EAE and *B*. *acidifaciens* significantly more abundant in RR-EAE; therefore, there may be a role for these bacteria in mediating different courses of EAE. Additionally, using LEfSe analysis, an algorithm for identifying biomarkers by emphasizing both statistical significance and consistent biological relevance, differentially abundant bacterial taxa were detected as discriminative for each naïve (naïve C57BL/6 and naïve SJL/J) and disease (CP-EAE and RR-EAE) state. Also, of note, we identified several taxa of bacteria present in our naïve and diseased mice that were also identified in a study evaluating the bidirectional association between the gut microbiota and EAE in the biphasic NOD model of disease^6^. Additionally, there were bacterial taxa identified in the NOD model of EAE (in both naïve and diseased mice) that were not detected in any of our groups, further strengthening a potential role for mouse strain in shaping microbiome composition and EAE pathogenesis^6^. It would be very interesting to evaluate the effect of enriching C57BL/6 mice with Firmicutes, specifically those of order *Clostridiales*, on the ability to mount an immunotolerant response characterized by T regs, and subsequent remission, similar to that seen in diseased SJL/J mice. Based on literature indicating that Clostridia from mouse and human intestines induce regulatory T cells, as well as correlates with T reg-induced remission in a T-synthase-deletion model of relapsing colitis, we speculate that this may shape an immune response that favors remission in the C57BL/6 mice^31,46,47^. Likewise, it would be interesting to determine if colonizing SJL/J mice with *A*. *muciniphila* or *Peptococceae rc4-4* can prevent a primary remission and/or promote a more chronic course of disease. Further studies manipulating the gut microbial composition in naïve C57BL/6 and SJL/J mice, as well as in CP-EAE and RR-EAE mice and evaluation of microbial metabolites in the form of short-chain fatty acids will more precisely identify the role of such microbiota in shaping the immune response and the clinical course of EAE.

## Materials and Methods

### Mice

Female, 8–10-week-old C57BL/6 or SJL/J mouse strains were purchased from The Jackson Laboratory (Bar Harbor, ME). Mice were maintained in conventional housing at Association for Assessment and Accreditation of Laboratory Animal Care (AALAC)–accredited University of South Carolina, School of Medicine Animal Facility in accordance with Institutional Animal Care and Use Committee (IACUC)-approved protocols and under National Institutes of Health guidelines.

### Reagents

Myelin oligodendrocyte peptide (MOG_35–55_) (H-MEVGWYRSPFSRVVHLYRNGK-OH) and myelin proteolipid protein peptide (PLP_139–151_) (H-HSLGKWLGHPDKF-OH) were from PolyPeptide Laboratories Sand Diego (San Diego, CA).

### EAE induction and Evaluation

EAE was induced in 8–10 week old female C57BL/6 mice as previously described^30^. Briefly, on day 0, mice were immunized via subcutaneous injection in each hind flank of 75 µg myelin oligodendrocyte peptide (MOG_35–55_)(for a total of 150 µg MOG_35–55_) emulsified in 50 µL complete Freund’s adjuvant (CFA) (Difco, Detroit, MI) containing 6 mg/mL killed *Mycobacterium tuberculosis* H37Ra (Difco). Two hours following immunization, mice were given 200 ng pertussis toxin (List Biologicals, Campbell, CA) via intraperitoneal injection, while 400 ng was given i.p. on day 2. EAE was induced in 8–10-week-old female SJL/J mice similarly; however, myelin proteolipid protein peptide (PLP_139–151_) was used to immunize these mice and 4 mg/mL *Mycobacterium tuberculosis* was added to CFA. Animals were monitored daily following immunization and symptoms of disease were evaluated and recorded as clinical scores based on the following criteria; 0 = no symptoms; 1 = partial loss of tail tonicity; 2 = complete tail atony, clumsy gait; 3 = hind limb weakness, partial paralysis; 4 = complete hind limb paralysis, fore limb weakness; and 5 = tetraplegia, moribund. Additional measures were taken to ensure accessibility to fresh food and water for paralytic animals. Mice that were moribund were euthanized by overdose of inhalant anesthetic isoflurane, as indicated in IACUC- and AALAS-approved protocols and death was not used as an index for clinical scores.

### Fecal Collection and DNA Isolation

Two weeks prior to disease induction, in order to rule out a shipment effect between strains and to account for inter-individual variability, four cages of each strain from the same shipment were randomized. Following two weeks of acclimation, mice were randomly selected from the four cages for disease induction or to remain naïve. Mice were then immunized, or not, and were separated into naïve or EAE cages for the remainder of the experiment. All mice were fed identical diets and housed under the same conditions, in adjacent positions on the rack. In order to account for any cage-effects, mice were randomly selected from each of the naïve and diseased cages for stool collection. On the same day, fecal pellets were collected from naïve mice and diseased mice (at the peak of disease, day 13 post-immunization), over a 12-hour period, with alternating 1-hour periods of collection and rest and stored immediately at −20 °C. Isolation of DNA from stool was performed using a QIAamp DNA Stool Mini Kit (Qiagen, Valencia, CA) according to manufacturer’s protocol.

### 16S rRNA Amplicon Sequencing and Analysis

16S V4 amplicons were generated according to 16S Metagenomic Sequencing Library Preparation Guide provided by Illumina Technologies. Briefly, The V4 hypervariable region of 16S rRNA was amplified from DNA isolated from feces. Primers specific for the V4 region^48^ were generated with the recommended overhang adapter sequences added (Forward overhang: 5′ TCGTCGGCAGCGTCAGATGTGTATAAGAGACAG and Reverse overhang: 5′ GTCTCGTGGGCTCGGAGATGTGTATAAGAGACAG) (Integrated DNA Technologies, Coralville, IA). Next, dual indices and Illumina sequencing adapters were added to amplicons using the Nextera XT Index Kit (Illumina, San Diego, CA). Libraries were pooled, denatured and spiked with PhiX internal control prior to being run on the Illumina MiSeq system using MiSeq Reagent Kit v3 (Illumina). FASTQ files generated by the MiSeq System were then analyzed using the National Institutes of Health’s microbiome analysis platform, Nephele (https://nephele.niaid.nih.gov/#home). Sequencing data was analyzed using QIIME FASTQ paired end pipeline with chimera removal and open-reference OTU picking with Silva 99. Nephele output provided alpha and beta diversity analysis and principle coordinate analyses, as well as heat maps and OTU tables and significance. Briefly, the OTU table was rarified to 81,094 sequences per sample and with-in sample diversity (α-diversity) metrics were generated using the Qiime pipeline in the Nephele platform: *Chao1*, observed OTUs, phylogenetic diversity, and Shannon index. Metrics, generated by Qiime, evaluating α-diversity were evaluated for significance using a one-way ANOVA with multiple comparisons, and Tukey’s post hoc analysis was performed to generate the reported adjusted p-values. In order to assess β-diversity, Qiime-calculated weighted and unweighted UniFrac distance matrices were used to run principle coordinate analyses and generate Emperor PCoA plots. Also using UniFrac distances, Qiime-produced boxplots were used in order to visualize within-group and between-group distances and statistical significance was assessed by two-sample t-tests for all pairs of boxplots, using Bonferroni correction to generate adjusted p-values. Furthermore, permutational multivariate analysis of variance using Qiime-generated UniFrac weighted and unweighted distance matrices was carried out with the adonis function in R vegan package with 1000 permutations. OTU data was used to calculate percent total OTUs to generate heat maps and individual bar graphs using Genesis and GraphPad Prism 7 software, respectively. Significance in bar charts for taxa-level analysis was assessed using one-way ANOVA with Tukey’s multiple comparisons test, and adjusted p-values reported.

### Biomarker Analysis

To identify possible OTU and KEGG biomarkers associated with each group, a linear discriminant effect size (LEfSe) analysis was performed using the Galaxy web application (http://huttenhower.sph.harvard.edu/galaxy/)^22^. Bacterial abundance profiles were calculated at taxonomic levels from phylum to species in percent abundance and alpha values ≥ 0.05 (Kruskal-Wallis test) and a logarithmic LDA score ≥ 3.0 were used as thresholds.

## Supplementary information

Supplementary Material

## Data Availability

The datasets generated and/or analyzed during the current study are included in this published article or are available from the corresponding author upon reasonable request. Microbiome datasets have been deposited into the NCBI SRA database and assigned accession code PRJNA525682.

## References

[1] Compston A, Coles A (2002). Multiple sclerosis. Lancet.

[2] Doshi A, Chataway J (2016). Multiple sclerosis, a treatable disease. Clin Med (Lond).

[3] Kirby, T.O. & Ochoa-Reparaz, J. The Gut Microbiome in Multiple Sclerosis: A Potential Therapeutic Avenue. *Med Sci* (*Basel*) **6**(3) (2018). 10.3390/medsci6030069 PMC6163724 30149548

[4] Miller SD, Karpus WJ (2007). Experimental autoimmune encephalomyelitis in the mouse. Curr Protoc Immunol.

[5] Fletcher JM (2010). T cells in multiple sclerosis and experimental autoimmune encephalomyelitis. Clin Exp Immunol.

[6] Colpitts SL (2017). A bidirectional association between the gut microbiota and CNS disease in a biphasic murine model of multiple sclerosis. Gut Microbes.

[7] Sawcer S (2011). The major cause of multiple sclerosis is environmental: genetics has a minor role–no. Mult Scler.

[8] Munoz-Culla M, Irizar H, Otaegui D (2013). The genetics of multiple sclerosis: review of current and emerging candidates. Appl Clin Genet.

[9] Brestoff JR, Artis D (2013). Commensal bacteria at the interface of host metabolism and the immune system. Nat Immunol.

[10] Rooks MG, Garrett WS (2016). Gut microbiota, metabolites and host immunity. Nat Rev Immunol.

[11] Maynard CL (2012). Reciprocal interactions of the intestinal microbiota and immune system. Nature.

[12] Ochoa-Reparaz J (2009). Role of gut commensal microflora in the development of experimental autoimmune encephalomyelitis. J Immunol.

[13] Lee YK (2011). Proinflammatory T-cell responses to gut microbiota promote experimental autoimmune encephalomyelitis. Proc Natl Acad Sci USA.

[14] Ochoa-Reparaz J (2010). A polysaccharide from the human commensal Bacteroides fragilis protects against CNS demyelinating disease. Mucosal Immunol.

[15] Stanisavljevic S (2016). Gut-associated lymphoid tissue, gut microbes and susceptibility to experimental autoimmune encephalomyelitis. Benef Microbes.

[16] Miyake S (2015). Dysbiosis in the Gut Microbiota of Patients with Multiple Sclerosis, with a Striking Depletion of Species Belonging to Clostridia XIVa and IV Clusters. PLoS One.

[17] Cantarel BL (2015). Gut microbiota in multiple sclerosis: possible influence of immunomodulators. J Investig Med.

[18] Bhargava P, Mowry EM (2014). Gut microbiome and multiple sclerosis. Curr Neurol Neurosci Rep.

[19] Jangi S (2016). Alterations of the human gut microbiome in multiple sclerosis. Nat Commun.

[20] Chitrala KN (2017). CD44 deletion leading to attenuation of experimental autoimmune encephalomyelitis results from alterations in gut microbiome in mice. Eur J Immunol.

[21] McGeachy MJ, Stephens LA, Anderton SM (2005). Natural recovery and protection from autoimmune encephalomyelitis: contribution of CD4+ CD25+ regulatory cells within the central nervous system. J Immunol.

[22] Segata N (2011). Metagenomic biomarker discovery and explanation. Genome Biol.

[23] Zozulya AL, Wiendl H (2008). The role of regulatory T cells in multiple sclerosis. Nat Clin Pract Neurol.

[24] Costantino CM, Baecher-Allan C, Hafler DA (2008). Multiple sclerosis and regulatory T cells. J Clin Immunol.

[25] Venken K (2008). Compromised CD4+ CD25(high) regulatory T-cell function in patients with relapsing-remitting multiple sclerosis is correlated with a reduced frequency of FOXP3-positive cells and reduced FOXP3 expression at the single-cell level. Immunology.

[26] Frisullo G (2009). Regulatory T cells fail to suppress CD4T+-bet+ T cells in relapsing multiple sclerosis patients. Immunology.

[27] Guan H, Nagarkatti PS, Nagarkatti M (2011). CD44 Reciprocally regulates the differentiation of encephalitogenic Th1/Th17 and Th2/regulatory T cells through epigenetic modulation involving DNA methylation of cytokine gene promoters, thereby controlling the development of experimental autoimmune encephalomyelitis. J Immunol.

[28] Guan H (2016). Inverse correlation of expression of microRNA-140-5p with progression of multiple sclerosis and differentiation of encephalitogenic T helper type 1 cells. Immunology.

[29] Rouse M, Nagarkatti M, Nagarkatti PS (2013). The role of IL-2 in the activation and expansion of regulatory T-cells and the development of experimental autoimmune encephalomyelitis. Immunobiology.

[30] Rouse M (2013). Indoles mitigate the development of experimental autoimmune encephalomyelitis by induction of reciprocal differentiation of regulatory T cells and Th17 cells. Br J Pharmacol.

[31] Atarashi K (2011). Induction of colonic regulatory T cells by indigenous Clostridium species. Science.

[32] Vital M, Howe AC, Tiedje JM (2014). Revealing the bacterial butyrate synthesis pathways by analyzing (meta)genomic data. MBio.

[33] Serino M (2012). Metabolic adaptation to a high-fat diet is associated with a change in the gut microbiota. Gut.

[34] Derrien M (2011). Modulation of Mucosal Immune Response, Tolerance, and Proliferation in Mice Colonized by the Mucin-Degrader Akkermansia muciniphila. Front Microbiol.

[35] Derrien M (2004). Akkermansia muciniphila gen. nov., sp. nov., a human intestinal mucin-degrading bacterium. Int J Syst Evol Microbiol.

[36] Ganesh BP (2013). Commensal Akkermansia muciniphila exacerbates gut inflammation in Salmonella Typhimurium-infected gnotobiotic mice. PLoS One.

[37] Peterson LW, Artis D (2014). Intestinal epithelial cells: regulators of barrier function and immune homeostasis. Nat Rev Immunol.

[38] Li, X. *et al*. Expanding xylose metabolism in yeast for plant cell wall conversion to biofuels. *Elife*, 4 (2015). 10.7554/eLife.05896 PMC4338637 25647728

[39] Plovier H (2017). A purified membrane protein from Akkermansia muciniphila or the pasteurized bacterium improves metabolism in obese and diabetic mice. Nat Med.

[40] Greer RL (2016). Akkermansia muciniphila mediates negative effects of IFNgamma on glucose metabolism. Nat Commun.

[41] Yamanaka T (2009). Gene expression profile and pathogenicity of biofilm-forming Prevotella intermedia strain 17. BMC Microbiol.

[42] Selvanantham T (2016). NKT Cell-Deficient Mice Harbor an Altered Microbiota That Fuels Intestinal Inflammation during Chemically Induced Colitis. J Immunol.

[43] Kim KA (2012). High fat diet-induced gut microbiota exacerbates inflammation and obesity in mice via the TLR4 signaling pathway. PLoS One.

[44] Jiang W (2015). Dysbiosis gut microbiota associated with inflammation and impaired mucosal immune function in intestine of humans with non-alcoholic fatty liver disease. Sci Rep.

[45] Kang CS (2013). Extracellular vesicles derived from gut microbiota, especially Akkermansia muciniphila, protect the progression of dextran sulfate sodium-induced colitis. PLoS One.

[46] Atarashi K (2013). Treg induction by a rationally selected mixture of Clostridia strains from the human microbiota. Nature.

[47] Jacobs JP (2017). Microbial, metabolomic, and immunologic dynamics in a relapsing genetic mouse model of colitis induced by T-synthase deficiency. Gut Microbes.

[48] Chassaing B (2015). Dietary emulsifiers impact the mouse gut microbiota promoting colitis and metabolic syndrome. Nature.

