# Supplementary material for: The role of gut microbiota in shaping the relapse-remitting and chronic-progressive forms of multiple sclerosis in mouse models
Source: Sci Rep. 2019 May 6;9:6923. doi: 10.1038/s41598-019-43356-7 (PMC6502871; doi:10.1038/s41598-019-43356-7)
Supplement: Supplementary file 1 — Supplementary Material [file 41598_2019_43356_MOESM1_ESM.pdf]

The role of gut microbiota in shaping the relapse-remitting and chronic-progressive forms of multiple sclerosis in mouse models

K. Alexa Orr Gandy, Jiajia Zhang, Prakash Nagarkatti, and Mitzi Nagarkatti

Supplementary Material

A)

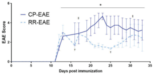

B)

Day v Day	Change in EAE Score		
13 v 16	-1.4	remit day 16	†
16 v 17	1.2	relapse day 17	‡
17 v 25	-1.5	remit day 25	†
25 v 30	1.3	relapse day 30	‡

Supplemental Figure S1

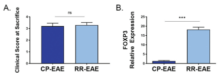

Supplemental Figure S2

		t statistic	p-value (Bonferroni-corrected)
All within TreatmentGroup	All between TreatmentGroup	-9.63	3.24E-14
All within TreatmentGroup	B6EAE vs. B6EAE	-0.26	1
All within TreatmentGroup	B6N vs. B6N	0.75	1
All within TreatmentGroup	SJLEAE vs. SJLEAE	-1.71	1
All within TreatmentGroup	SJLN vs. SJLN	1.24	1
All within TreatmentGroup	B6EAE vs. B6N	-5.24	0.0003
All within TreatmentGroup	B6EAE vs. SJLEAE	-8.62	2.34E-08
All within TreatmentGroup	B6EAE vs. SJLN	-10.21	3.24E-10
All within TreatmentGroup	B6N vs. SJLEAE	-8.77	3.34E-08
All within TreatmentGroup	B6N vs. SJLN	-6.11	5.28E-05
All within TreatmentGroup	SJLEAE vs. SJLN	-8.89	5.93E-08
All between TreatmentGroup	B6EAE vs. B6EAE	6.65	1.39E-07
All between TreatmentGroup	B6N vs. B6N	6.18	1.31E-06
All between TreatmentGroup	SJLEAE vs. SJLEAE	2.13	1
All between TreatmentGroup	SJLN vs. SJLN	5.07	0.0001
All between TreatmentGroup	B6EAE vs. B6N	4.30	0.0026
All between TreatmentGroup	B6EAE vs. SJLEAE	-1.27	1
All between TreatmentGroup	B6EAE vs. SJLN	-1.01	1
All between TreatmentGroup	B6N vs. SJLEAE	-2.32	1
All between TreatmentGroup	B6N vs. SJLN	1.91	1
All between TreatmentGroup	SJLEAE vs. SJLN	-2.84	0.3677
B6EAE vs. B6EAE	B6N vs. B6N	0.98	1
B6EAE vs. B6EAE	SJLEAE vs. SJLEAE	-1.53	1
B6EAE vs. B6EAE	SJLN vs. SJLN	1.54	1
B6EAE vs. B6EAE	B6EAE vs. B6N	-4.56	0.0060
B6EAE vs. B6EAE	B6EAE vs. SJLEAE	-7.24	1.50E-05
B6EAE vs. B6EAE	B6EAE vs. SJLN	-10.66	1.48E-08
B6EAE vs. B6EAE	B6N vs. SJLEAE	-7.38	2.63E-05
B6EAE vs. B6EAE	B6N vs. SJLN	-6.17	0.0003
B6EAE vs. B6EAE	SJLEAE vs. SJLN	-7.90	2.86E-05
B6N vs. B6N	SJLEAE vs. SJLEAE	-1.97	1
B6N vs. B6N	SJLN vs. SJLN	0.76	1
B6N vs. B6N	B6EAE vs. B6N	-5.36	0.0011
B6N vs. B6N	B6EAE vs. SJLEAE	-7.07	6.60E-05
B6N vs. B6N	B6EAE vs. SJLN	-12.33	1.08E-08
B6N vs. B6N	B6N vs. SJLEAE	-7.06	0.0001
B6N vs. B6N	B6N vs. SJLN	-7.52	8.08E-05
B6N vs. B6N	SJLEAE vs. SJLN	-7.70	0.0002
SJLEAE vs. SJLEAE	SJLN vs. SJLN	2.04	1
SJLEAE vs. SJLEAE	B6EAE vs. B6N	-0.59	1
SJLEAE vs. SJLEAE	B6EAE vs. SJLEAE	-2.65	1
SJLEAE vs. SJLEAE	B6EAE vs. SJLN	-4.27	0.0387
SJLEAE vs. SJLEAE	B6N vs. SJLEAE	-2.88	0.8480
SJLEAE vs. SJLEAE	B6N vs. SJLN	-1.69	1
SJLEAE vs. SJLEAE	SJLEAE vs. SJLN	-3.24	0.5806
SJLN vs. SJLN	B6EAE vs. B6N	-5.29	0.0019
SJLN vs. SJLN	B6EAE vs. SJLEAE	-6.07	0.0010
SJLN vs. SJLN	B6EAE vs. SJLN	-13.74	1.88E-08
SJLN vs. SJLN	B6N vs. SJLEAE	-5.96	0.0031
SJLN vs. SJLN	B6N vs. SJLN	-8.49	7.67E-05
SJLN vs. SJLN	SJLEAE vs. SJLN	-6.64	0.0038
B6EAE vs. B6N	B6EAE vs. SJLEAE	-5.37	0.0004
B6EAE vs. B6N	B6EAE vs. SJLN	-7.36	1.25E-06
B6EAE vs. B6N	B6N vs. SJLEAE	-6.09	7.23E-05
B6EAE vs. B6N	B6N vs. SJLN	-2.26	1
B6EAE vs. B6N	SJLEAE vs. SJLN	-6.87	1.46E-05
B6EAE vs. SJLEAE	B6EAE vs. SJLN	0.36	1
B6EAE vs. SJLEAE	B6N vs. SJLEAE	-1.00	1
B6EAE vs. SJLEAE	B6N vs. SJLN	3.25	0.2171
B6EAE vs. SJLEAE	SJLEAE vs. SJLN	-1.65	1
B6EAE vs. SJLN	B6N vs. SJLEAE	-1.64	1
B6EAE vs. SJLN	B6N vs. SJLN	5.72	0.0003
B6EAE vs. SJLN	SJLEAE vs. SJLN	-2.72	0.8336
B6N vs. SJLEAE	B6N vs. SJLN	4.06	0.0343
B6N vs. SJLEAE	SJLEAE vs. SJLN	-0.63	1
B6N vs. SJLN	SJLEAE vs. SJLN	-5.06	0.0046

Supplemental Table S3. Pairwise Comparison of Unweighted UniFrac Distances Within and Between Groups Two-sample t-tests were performed on all pairs of distance boxplots to assess significance, with Bonferroni post hoc analysis used to generate adjusted p-values, reported here.

		t statistic	p-value (Bonferroni-corrected)
All within TreatmentGroup	All between TreatmentGroup	-6.02	1.80E-06
All within TreatmentGroup	B6EAE vs. B6EAE	-1.81	1
All within TreatmentGroup	B6N vs. B6N	2.42	1
All within TreatmentGroup	SJLEAE vs. SJLEAE	-0.17	1
All within TreatmentGroup	SJLN vs. SJLN	0.69	1
All within TreatmentGroup	B6EAE vs. B6N	-3.33	0.1253
All within TreatmentGroup	B6EAE vs. SJLEAE	-3.64	0.0583
All within TreatmentGroup	B6EAE vs. SJLN	-6.06	4.22E-05
All within TreatmentGroup	B6N vs. SJLEAE	-1.52	1
All within TreatmentGroup	B6N vs. SJLN	-3.88	0.0324
All within TreatmentGroup	SJLEAE vs. SJLN	-4.80	0.0029
All between TreatmentGroup	B6EAE vs. B6EAE	1.78	1
All between TreatmentGroup	B6N vs. B6N	7.10	2.13E-08
All between TreatmentGroup	SJLEAE vs. SJLEAE	2.46	1
All between TreatmentGroup	SJLN vs. SJLN	3.63	0.0319
All between TreatmentGroup	B6EAE vs. B6N	1.66	1
All between TreatmentGroup	B6EAE vs. SJLEAE	-0.25	1
All between TreatmentGroup	B6EAE vs. SJLN	-2.71	0.5280
All between TreatmentGroup	B6N vs. SJLEAE	3.22	0.1149
All between TreatmentGroup	B6N vs. SJLN	-0.01	1
All between TreatmentGroup	SJLEAE vs. SJLN	-2.32	1
B6EAE vs. B6EAE	B6N vs. B6N	4.00	0.0874
B6EAE vs. B6EAE	SJLEAE vs. SJLEAE	0.98	1
B6EAE vs. B6EAE	SJLN vs. SJLN	1.79	1
B6EAE vs. B6EAE	B6EAE vs. B6N	-0.56	1
B6EAE vs. B6EAE	B6EAE vs. SJLEAE	-1.26	1
B6EAE vs. B6EAE	B6EAE vs. SJLN	-3.29	0.2125
B6EAE vs. B6EAE	B6N vs. SJLEAE	0.77	1
B6EAE vs. B6EAE	B6N vs. SJLN	-1.49	1
B6EAE vs. B6EAE	SJLEAE vs. SJLN	-2.62	1
B6N vs. B6N	SJLEAE vs. SJLEAE	-14.02	0.0001
B6N vs. B6N	SJLN vs. SJLN	-5.08	0.0946
B6N vs. B6N	B6EAE vs. B6N	-7.43	7.54E-06
B6N vs. B6N	B6EAE vs. SJLEAE	-5.42	0.0021
B6N vs. B6N	B6EAE vs. SJLN	-13.28	3.04E-09
B6N vs. B6N	B6N vs. SJLEAE	-11.11	4.09E-07
B6N vs. B6N	B6N vs. SJLN	-16.33	1.40E-09
B6N vs. B6N	SJLEAE vs. SJLN	-9.71	1.68E-05
SJLEAE vs. SJLEAE	SJLN vs. SJLN	2.67	1
SJLEAE vs. SJLEAE	B6EAE vs. B6N	-2.13	1
SJLEAE vs. SJLEAE	B6EAE vs. SJLEAE	-1.91	1
SJLEAE vs. SJLEAE	B6EAE vs. SJLN	-5.49	0.0033
SJLEAE vs. SJLEAE	B6N vs. SJLEAE	-1.88	1
SJLEAE vs. SJLEAE	B6N vs. SJLN	-5.53	0.0064
SJLEAE vs. SJLEAE	SJLEAE vs. SJLN	-4.03	0.1583
SJLN vs. SJLN	B6EAE vs. B6N	-3.53	0.1325
SJLN vs. SJLN	B6EAE vs. SJLEAE	-2.77	0.9043
SJLN vs. SJLN	B6EAE vs. SJLN	-7.12	0.0002
SJLN vs. SJLN	B6N vs. SJLEAE	-4.35	0.0519
SJLN vs. SJLN	B6N vs. SJLN	-7.83	0.0002
SJLN vs. SJLN	SJLEAE vs. SJLN	-5.19	0.0268
B6EAE vs. B6N	B6EAE vs. SJLEAE	-1.30	1
B6EAE vs. B6N	B6EAE vs. SJLN	-4.39	0.0074
B6EAE vs. B6N	B6N vs. SJLEAE	2.15	1
B6EAE vs. B6N	B6N vs. SJLN	-1.58	1
B6EAE vs. B6N	SJLEAE vs. SJLN	-3.55	0.0949
B6EAE vs. SJLEAE	B6EAE vs. SJLN	-1.71	1
B6EAE vs. SJLEAE	B6N vs. SJLEAE	2.55	1
B6EAE vs. SJLEAE	B6N vs. SJLN	0.17	1
B6EAE vs. SJLEAE	SJLEAE vs. SJLN	-1.46	1
B6EAE vs. SJLN	B6N vs. SJLEAE	7.63	3.63E-06
B6EAE vs. SJLN	B6N vs. SJLN	3.29	0.1945
B6EAE vs. SJLN	SJLEAE vs. SJLN	-0.25	1
B6N vs. SJLEAE	B6N vs. SJLN	-5.50	0.0010
B6N vs. SJLEAE	SJLEAE vs. SJLN	-5.86	0.0008
B6N vs. SJLN	SJLEAE vs. SJLN	-2.68	0.9697

Supplemental Table S4. Pairwise Comparison of Weighted UniFrac Distances Within and Between Groups. Two-sample t-tests were performed on each pair of distance boxplots to assess significance, with Bonferroni post-hoc analysis used to generate adjusted p-values, reported here.

	Naïve C57BL/6	CP-EAE	Naïve SJL/J	RR-EAE
p__Bacteroidetes	67.70 ± 1.37	56.30 ± 7.57	28.17 ± 4.53	71.07 ± 5.40
p__Firmicutes	31.98 ± 1.28	34.34 ± 5.13	71.70 ± 4.44	27.63 ± 5.08
p__Tenericutes	0.20 ± 0.04	0.52 ± 0.12	0.10 ± 0.1	1.23 ± 0.52
p__Verrucomicrobia	0.08 ± 0.08	8.82 ± 2.84	0 ± 0	0 ± 0

Supplemental Table S5. Phylum level taxonomic composition of samples.
Values represent mean percent abundance ± SEM. p, phylum

	Naïve C57BL/6	CP-EAE	Naïve SJL/J	RR-EAE
p__Bacteroidetes;c__Bacteroidia	67.70 ± 1.37	56.30 ± 7.57	28.17 ± 4.53	71.07 ± 5.4
p__Firmicutes;c__Bacilli	0.60 ± 0.04	0.16 ± 0.07	0.03 ± 0.03	0.10 ± 0
p__Firmicutes;c__Clostridia	31.33 ± 1.27	34.10 ± 5.14	71.63 ± 4.47	27.57 ± 5.06
p__Tenericutes;c__Mollicutes	0.20 ± 0.04	0.52 ± 0.12	0.10 ± 0.10	1.23 ± 0.52
p__Verrucomicrobia;c__Verrucomicrobiae	0.08 ± 0.08	8.82 ± 2.84	0 ± 0	0 ± 0

Supplemental Table S6. Class level taxonomic composition of samples.
Values represent mean percent abundance ± SEM. p, phylum; c, class

	Naïve C57BL/6	CP-EAE	Naïve SJL/J	RR-EAE
p__Bacteroidetes;c__Bacteroidia;o__Bacteroidales	67.70± 1.37	56.30± 7.57	28.17± 4.53	71.07± 5.4
p__Firmicutes;c__Bacilli;o__Lactobacillales	0.53± 0.05	0.08± 0.04	0.03± 0.03	0.10± 0
p__Firmicutes;c__Bacilli;o__Turicibacterales	0.08± 0.03	0.12± 0.05	0± 0	0± 0
p__Firmicutes;c__Clostridia;o__Clostridiales	31.33± 1.27	34.10± 5.14	71.63± 4.47	27.57± 5.06
p__Tenericutes;c__Mollicutes;o__Anaeroplasmatales	0.10± 0.04	0.22± 0.07	0.10± 0.1	0.30± 0.17
p__Tenericutes;c__Mollicutes;o__RF39	0.10± 0	0.32± 0.06	0± 0	0.93± 0.38
p__Verrucomicrobia;c__Verrucomicrobiae;o__Verrucomicrobiales	0.08± 0.08	8.82± 2.84	0± 0	0± 0

Supplemental Table S7. Order level taxonomic composition of samples.
Values represent mean percent abundance ± SEM. p, phylum; c, class; o, order

	Naïve C57BL/6	CP-EAE	Naïve SJL/J	RR-EAE
p__Bacteroidetes;c__Bacteroidia;o__Bacteroidales;f__Bacteroidaceae	0 ± 0	0 ± 0	0 ± 0	5.13 ± 2.24
p__Bacteroidetes;c__Bacteroidia;o__Bacteroidales;f__Porphyromonadaceae	0 ± 0	0 ± 0	0 ± 0	0.97 ± 0.18
p__Bacteroidetes;c__Bacteroidia;o__Bacteroidales;f__Prevotellaceae	0 ± 0	0 ± 0	0 ± 0	4.13 ± 1.75
p__Bacteroidetes;c__Bacteroidia;o__Bacteroidales;f__Rikenellaceae	0 ± 0	0.12 ± 0.06	0 ± 0	1.53 ± 0.28
p__Bacteroidetes;c__Bacteroidia;o__Bacteroidales;f__S24-7	67.68 ± 1.34	56.14 ± 7.54	28.17 ± 4.53	58.47 ± 5.82
p__Bacteroidetes;c__Bacteroidia;o__Bacteroidales;f__[Odoribacteraceae]	0 ± 0	0 ± 0	0 ± 0	0.83 ± 0.33
p__Firmicutes;c__Bacilli;o__Lactobacillales;f__Lactobacillaceae	0.53 ± 0.05	0.04 ± 0.04	0.03 ± 0.03	0.10 ± 0
p__Firmicutes;c__Bacilli;o__Turicibacterales;f__Turicibacteraceae	0.08 ± 0.03	0.12 ± 0.049	0 ± 0	0 ± 0
p__Firmicutes;c__Clostridia;o__Clostridiales;f__	17.83 ± 1.03	23.98 ± 3.34	46.10 ± 2.37	20.33 ± 3.87
p__Firmicutes;c__Clostridia;o__Clostridiales;f__Clostridiaceae	0.23 ± 0.03	0.1 ± 0.05	0.10 ± 0	0.07 ± 0.03
p__Firmicutes;c__Clostridia;o__Clostridiales;f__Dehalobacteriaceae	0.23 ± 0.03	0.14 ± 0.04	0.23 ± 0.03	0.20 ± 0
p__Firmicutes;c__Clostridia;o__Clostridiales;f__Lachnospiraceae	6.93 ± 0.28	2.30 ± 0.5	8.80 ± 0.36	2.60 ± 0.35
p__Firmicutes;c__Clostridia;o__Clostridiales;f__Peptococcaceae	0 ± 0	0.90 ± 0.15	0 ± 0	0 ± 0
p__Firmicutes;c__Clostridia;o__Clostridiales;f__Ruminococcaceae	6.05 ± 0.55	6.68 ± 1.26	16.47 ± 2.28	4.33 ± 0.83
p__Tenericutes;c__Mollicutes;o__Anaeroplasmatales;f__Anaeroplasmataceae	0.10 ± 0.04	0.22 ± 0.07	0.10 ± 0.10	0.30±0.17
p__Tenericutes;c__Mollicutes;o__RF39;f__	0.1 ± 0	0.32 ± 0.06	0 ± 0	0.93 ± 0.38
p__Verrucomicrobia;c__Verrucomicrobiae;o__Verrucomicrobiales;f__Verrucomicrobiaceae	0.08 ± 0.08	8.82 ± 2.84	0 ± 0	0 ± 0

Supplemental Table S8. Family level taxonomic composition of samples. Values represent mean percent abundance ± SEM. p, phylum; c, class; o, order; f, family. Blank/unnamed taxa = unclassified.

	Naïve C57BL/6	CP-EAE	Naïve SJL/J	RR-EAE
p__Bacteroidetes;c__Bacteroidia;o__Bacteroidales;f__Bacteroidaceae;g__Bacteroides	0± 0	0± 0	0± 0	5.13± 2.24
p__Bacteroidetes;c__Bacteroidia;o__Bacteroidales;f__Porphyromonadaceae;g__Parabacteroides	0± 0	0± 0	0± 0	0.97± 0.18
p__Bacteroidetes;c__Bacteroidia;o__Bacteroidales;f__Prevotellaceae;g__Prevotella	0± 0	0± 0	0± 0	4.13± 1.75
p__Bacteroidetes;c__Bacteroidia;o__Bacteroidales;f__Rikenellaceae;g__	0± 0	0.12± 0.06	0± 0	1.53± 0.28
p__Bacteroidetes;c__Bacteroidia;o__Bacteroidales;f__S24-7;g__	67.68± 1.34	56.14± 7.54	28.17± 4.53	58.47± 5.82
p__Bacteroidetes;c__Bacteroidia;o__Bacteroidales;f__[Odoribacteraceae];g__Odoribacter	0.00± 0	0.00± 0	0.00± 0	0.83± 0.33
p__Firmicutes;c__Bacilli;o__Lactobacillales;f__Lactobacillaceae;g__Lactobacillus	0.53± 0.05	0.04± 0.04	0.03± 0.03	0.10± 0
p__Firmicutes;c__Bacilli;o__Turicibacterales;f__Turicibacteraceae;g__Turicibacter	0.08± 0.03	0.12± 0.05	0.00± 0	0.00± 0
p__Firmicutes;c__Clostridia;o__Clostridiales;f__;g__	17.83± 1.03	23.98± 3.34	46.10± 2.37	20.33± 3.87
p__Firmicutes;c__Clostridia;o__Clostridiales;f__Clostridiaceae;g__	0.10± 0	0.00± 0	0.10± 0	0.07± 0.03
p__Firmicutes;c__Clostridia;o__Clostridiales;f__Clostridiaceae;g__Clostridium	0.15± 0.03	0.04± 0.02	0.00± 0	0.00± 0
p__Firmicutes;c__Clostridia;o__Clostridiales;f__Dehalobacteriaceae;g__Dehalobacterium	0.23± 0.03	0.14± 0.04	0.23± 0.03	0.20± 0
p__Firmicutes;c__Clostridia;o__Clostridiales;f__Lachnospiraceae;g__	4.63± 0.35	1.50± 0.28	7.50± 0.67	2.00± 0.21
p__Firmicutes;c__Clostridia;o__Clostridiales;f__Lachnospiraceae;g__Anaerostipes	0.48± 0.09	0.20± 0.11	0.00± 0	0.00± 0
p__Firmicutes;c__Clostridia;o__Clostridiales;f__Lachnospiraceae;g__Coprococcus	0.20± 0	0.10± 0	0.50± 0.06	0.13± 0.03
p__Firmicutes;c__Clostridia;o__Clostridiales;f__Lachnospiraceae;g__Dorea	0.78± 0.03	0.00± 0	0.03± 0.03	0.10± 0.06
p__Firmicutes;c__Clostridia;o__Clostridiales;f__Lachnospiraceae;g__[Ruminococcus]	0.75± 0.06	0.48± 0.15	0.73± 0.24	0.33± 0.09
p__Firmicutes;c__Clostridia;o__Clostridiales;f__Peptococcaceae;g__rc4-4	0.00± 0	0.90± 0.15	0.00± 0	0.00± 0
p__Firmicutes;c__Clostridia;o__Clostridiales;f__Ruminococcaceae;g__	2.45± 0.18	2.48± 0.42	6.20± 2.07	2.10± 0.31
p__Firmicutes;c__Clostridia;o__Clostridiales;f__Ruminococcaceae;g__Oscillospira	2.88± 0.33	3.02± 0.67	8.50±0.23	1.97± 0.47
p__Firmicutes;c__Clostridia;o__Clostridiales;f__Ruminococcaceae;g__Ruminococcus	0.73± 0.17	1.18± 0.2	1.77± 0.09	0.27± 0.07
p__Tenericutes;c__Mollicutes;o__Anaeroplasmatales;f__Anaeroplasmataceae;g__Anaeroplasma	0.10± 0.04	0.22± 0.07	0.10± 0.1	0.30± 0.17
p__Tenericutes;c__Mollicutes;o__RF39;f__;g__	0.10± 0	0.32± 0.06	0.00± 0	0.93± 0.38
p__Verrucomicrobia;c__Verrucomicrobiae;o__Verrucomicrobiales;f__Verrucomicrobiaceae;g__Akkermansia	0.08± 0.08	8.82± 2.84	0.00± 0	0.00± 0

Supplemental Table S9. Genus level taxonomic composition of samples. Values represent mean percent abundance ± SEM. p, phylum; c, class; o, order; f, family; g, genus. Blank/unnamed taxa = unclassified.

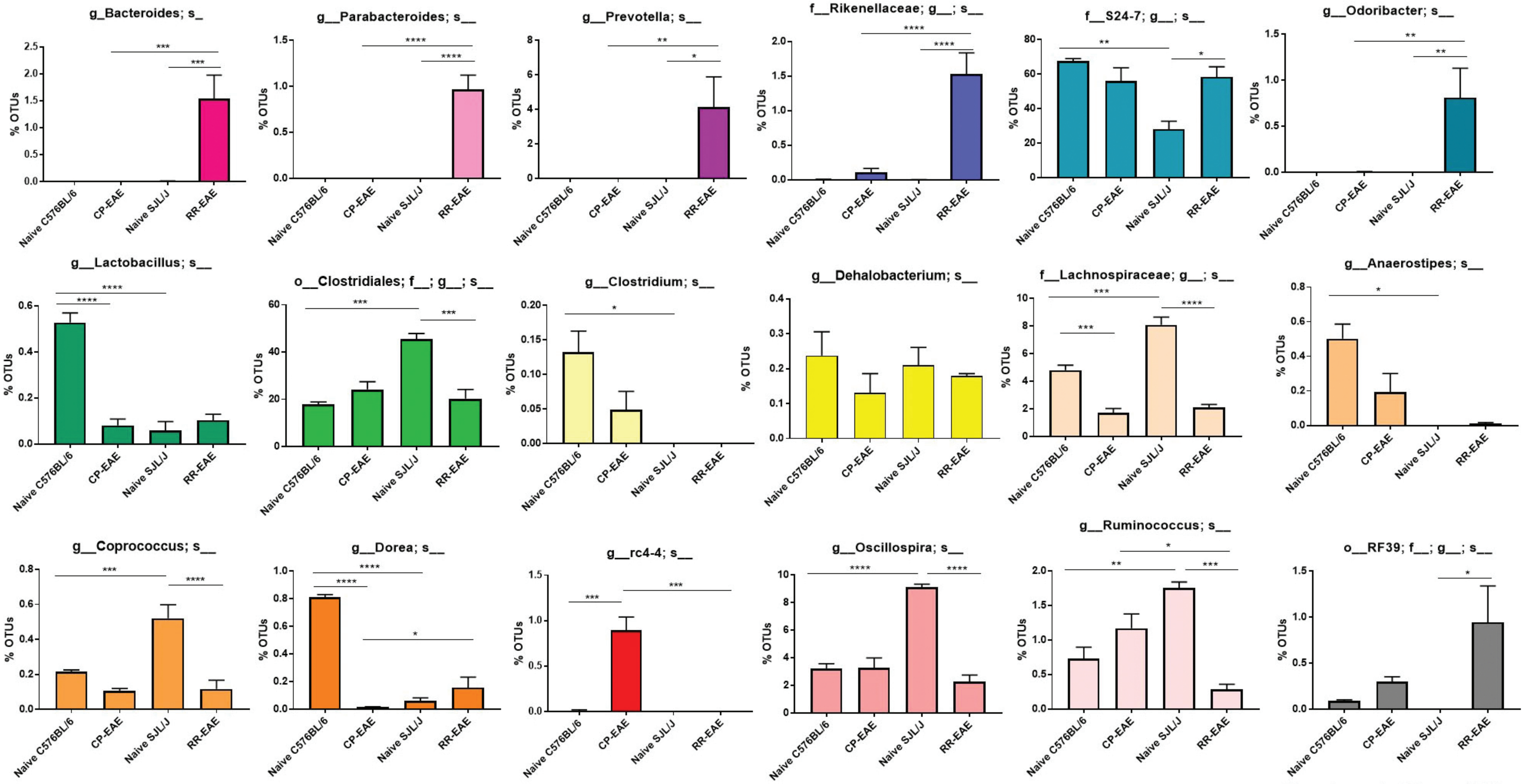

Supplemental Figure S10

	Naïve C57BL/6	CP-EAE	Naïve SJL/J	RR-EAE
p__Bacteroidetes; c__Bacteroidia; o__Bacteroidales; f__Bacteroidaceae; g__Bacteroides; s__	0±0	0± 0	0.01± 0	1.54± 0.76
p__Bacteroidetes; c__Bacteroidia; o__Bacteroidales; f__Bacteroidaceae; g__Bacteroides; s__acidifaciens	0±0	0± 0	0± 0	3.56± 3.11
p__Bacteroidetes; c__Bacteroidia; o__Bacteroidales; f__Porphyromonadaceae; g__Parabacteroides; s__	0±0	0± 0	0± 0	0.97± 0.27
p__Bacteroidetes; c__Bacteroidia; o__Bacteroidales; f__Prevotellaceae; g__Prevotella; s__	0±0	0± 0	0.01± 0.00	4.12± 3.06
p__Bacteroidetes; c__Bacteroidia; o__Bacteroidales; f__Rikenellaceae; g__; s__	0.01±0.00	0.11± 0.05	0± 0	1.53± 0.53
p__Bacteroidetes; c__Bacteroidia; o__Bacteroidales; f__S24-7; g__; s__	67.68±1.35	56.16± 7.53	28.17± 4.51	58.46± 10.1
p__Bacteroidetes; c__Bacteroidia; o__Bacteroidales; f__[Odoribacteraceae]; g__Odoribacter; s__	0±0	0.01± 0.00	0± 0	0.81± 0.55
p__Firmicutes; c__Bacilli; o__Lactobacillales; f__Lactobacillaceae; g__Lactobacillus; s__	0.53±0.04	0.08± 0.03	0.06± 0.04	0.10± 0.04
k__Bacteria; p__Firmicutes; c__Bacilli; o__Turicibacterales; f__Turicibacteraceae; g__Turicibacter; s__	0.07± 0.01	0.11± 0.04	0± 0	0± 0
p__Firmicutes; c__Clostridia; o__Clostridiales; f__; g__; s__	17.83±1.07	24.18± 3.3	45.49± 2.38	20.30± 6.69
k__Bacteria; p__Firmicutes; c__Clostridia; o__Clostridiales; f__Clostridiaceae; g__; s__	0.08± 0.00	0.04± 0.00	0.07± 0.01	0.08± 0.03
p__Firmicutes; c__Clostridia; o__Clostridiales; f__Clostridiaceae; g__Clostridium; s__	0.13±0.03	0.05± 0.03	0± 0	0± 0
p__Firmicutes; c__Clostridia; o__Clostridiales; f__Dehalobacteriaceae; g__Dehalobacterium; s__	0.24±0.03	0.13± 0.02	0.21± 0.03	0.18± 0.00
p__Firmicutes; c__Clostridia; o__Clostridiales; f__Lachnospiraceae; g__; s__	4.81±0.37	1.70± 0.33	8.09± 0.56	2.12± 0.37
p__Firmicutes; c__Clostridia; o__Clostridiales; f__Lachnospiraceae; g__Anaerostipes; s__	0.50±0.08	0.19± 0.11	0± 0.00	0.01± 0.01
p__Firmicutes; c__Clostridia; o__Clostridiales; f__Lachnospiraceae; g__Coprococcus; s__	0.22±0.01	0.11± 0.01	0.52± 0.08	0.12± 0.09
p__Firmicutes; c__Clostridia; o__Clostridiales; f__Lachnospiraceae; g__Dorea; s__	0.81±0.02	0.02± 0.00	0.06± 0.02	0.16± 0.13
p__Firmicutes; c__Clostridia; o__Clostridiales; f__Lachnospiraceae; g__[Ruminococcus]; s__gnavus	0.73±0.054	0.28± 0.08	0.70± 0.22	0.23± 0.08
p__Firmicutes; c__Clostridia; o__Clostridiales; f__Peptococcaceae; g__rc4-4; s__	0.01± 0.01	0.90± 0.14	0± 0	0± 0
p__Firmicutes; c__Clostridia; o__Clostridiales; f__Ruminococcaceae; g__; s__	2.11± 0.16	2.23± 0.37	5.61± 2.04	1.77± 0.49
p__Firmicutes; c__Clostridia; o__Clostridiales; f__Ruminococcaceae; g__Oscillospira; s__	3.23± 0.34	3.28± 0.72	9.11± 0.24	2.27± 0.83
p__Firmicutes; c__Clostridia; o__Clostridiales; f__Ruminococcaceae; g__Ruminococcus; s__	0.73± 0.16	1.17± 0.21	1.76± 0.08	0.29± 0.12
p__Tenericutes; c__Mollicutes; o__Anaeroplasmatales; f__Anaeroplasmataceae; g__Anaeroplasma; s__	0.12± 0.04	0.22± 0.08	0.09± 0.08	0.31± 0.31
p__Tenericutes; c__Mollicutes; o__RF39; f__; g__; s__	0.09± 0.01	0.30± 0.06	0± 0	0.95± 0.68
p__Verrucomicrobia; c__Verrucomicrobiae; o__Verrucomicrobiales; f__Verrucomicrobiaceae; g__Akkermansia; s__muciniphila	0.07± 0.07	8.82± 2.83	0± 0	0± 0.00

Supplemental Table S11. Species level taxonomic composition of samples. Values represent mean percent abundance ± SEM. p, phylum; c, class; o, order; f, family; g, genus; s, species. Blank/unnamed taxa = unclassified.

Comparison	B6N Bacterial taxa	LDA	CPEAE Bacterial taxa	LDA	SJLN Bacterial taxa	LDA	RREAE Bacterial taxa	LDA
B6N v CPEAE v SJLN v RREAE	Bacteria.Firmicutes.Bacilli	3.42	Bacteria.Firmicutes.Clostridia.Clostridiales.Lachnospiraceae.rc4_4	3.64	Bacteria.Cyanobacteria.4C0d_2	3.12	Bacteria.Bacteroidetes.Bacteroidia.Bacteroidales._Odoribacteraceae_	3.64
	Bacteria.Firmicutes.Bacilli.Lactobacillales	3.37	Bacteria.Firmicutes.Clostridia.Clostridiales.Lachnospiraceae.rc4_4_s_	3.64	Bacteria.Cyanobacteria.4C0d_2.YS2	3.12	Bacteria.Bacteroidetes.Bacteroidia.Bacteroidales._Odoribacteraceae_.Odoribacter	3.64
	Bacteria.Firmicutes.Bacilli.Lactobacillales.Lactobacillaceae	3.36	Bacteria.Firmicutes.Clostridia.Clostridiales.Peptococcaceae	3.64	Bacteria.Cyanobacteria.4C0d_2.YS2_f_	3.12	Bacteria.Bacteroidetes.Bacteroidia.Bacteroidales._Odoribacteraceae_.Odoribacter_s_	3.61
	Bacteria.Firmicutes.Bacilli.Lactobacillales.Lactobacillaceae.Lactobacillus	3.37	Bacteria.Verrucomicrobia	4.62	Bacteria.Cyanobacteria.4C0d_2.YS2_f_g_	3.11	Bacteria.Bacteroidetes.Bacteroidia.Bacteroidales.Bacteroidaceae	4.45
	Bacteria.Firmicutes.Bacilli.Lactobacillales.Lactobacillaceae.Lactobacillus_s_	3.39	Bacteria.Verrucomicrobia.Verrucomicrobiae	4.62	Bacteria.Cyanobacteria.4C0d_2.YS2_f_g_s_	3.09	Bacteria.Bacteroidetes.Bacteroidia.Bacteroidales.Bacteroidaceae.Bacteroides	4.44
	Bacteria.Firmicutes.Clostridia.Clostridiales.Lachnospiraceae._Ruminococcus_gnavus	3.38	Bacteria.Verrucomicrobia.Verrucomicrobiae.Verrucomicrobiales	4.62	Bacteria.Firmicutes.Clostridia.Clostridiales.Lachnospiraceae	4.54	Bacteria.Bacteroidetes.Bacteroidia.Bacteroidales.Bacteroidaceae.Bacteroides.acidifaciens	4.26
	Bacteria.Firmicutes.Clostridia.Clostridiales.Lachnospiraceae.Anaerostipes_s_	3.37	Bacteria.Verrucomicrobia.Verrucomicrobiae.Verrucomicrobiales.Verrucomicrobiaceae	4.62	Bacteria.Firmicutes.Clostridia.Clostridiales.Lachnospiraceae.Coprococcus	3.31	Bacteria.Bacteroidetes.Bacteroidia.Bacteroidales.Bacteroidaceae.Bacteroides_s_	3.92
	Bacteria.Firmicutes.Clostridia.Clostridiales.Lachnospiraceae.Dorea	3.61	Bacteria.Verrucomicrobia.Verrucomicrobiae.Verrucomicrobiales.Verrucomicrobiaceae.Akkermansia	4.62	Bacteria.Firmicutes.Clostridia.Clostridiales.Lachnospiraceae.Coprococcus_s_	3.30	Bacteria.Bacteroidetes.Bacteroidia.Bacteroidales.Porphyromonadaceae	3.71
	Bacteria.Firmicutes.Clostridia.Clostridiales.Lachnospiraceae.Dorea_s_	3.60	Bacteria.Verrucomicrobia.Verrucomicrobiae.Verrucomicrobiales.Verrucomicrobiaceae.Akkermansia.muciniphila	4.62	Bacteria.Firmicutes.Clostridia.Clostridiales.Lachnospiraceae_g_	4.47	Bacteria.Bacteroidetes.Bacteroidia.Bacteroidales.Porphyromonadaceae.Parabacteroides	3.72
					Bacteria.Firmicutes.Clostridia.Clostridiales.Lachnospiraceae_g_s_	4.52	Bacteria.Bacteroidetes.Bacteroidia.Bacteroidales.Porphyromonadaceae.Parabacteroides_s_	3.70
					Bacteria.Firmicutes.Clostridia.Clostridiales.Ruminococcaceae	4.75	Bacteria.Bacteroidetes.Bacteroidia.Bacteroidales.Prevotellaceae.Prevotella_s_	4.36
					Bacteria.Firmicutes.Clostridia.Clostridiales.Ruminococcaceae.Oscillospira	4.52	Bacteria.Bacteroidetes.Bacteroidia.Bacteroidales.Rikenellaceae_g_	3.88
					Bacteria.Firmicutes.Clostridia.Clostridiales.Ruminococcaceae.Oscillospira_s_	4.54	Bacteria.Bacteroidetes.Bacteroidia.Bacteroidales.Rikenellaceae_g_s_	3.87
					Bacteria.Firmicutes.Clostridia.Clostridiales.Ruminococcaceae.Ruminococcus	3.87	Bacteria.Tenericutes.Mollicutes.RF39	3.71
					Bacteria.Firmicutes.Clostridia.Clostridiales.Ruminococcaceae.Ruminococcus_s_	3.87	Bacteria.Tenericutes.Mollicutes.RF39_f_	3.72
					Bacteria.Proteobacteria.Alphaproteobacteria.RF32	3.17	Bacteria.Tenericutes.Mollicutes.RF39_f_g_	3.69
					Bacteria.Proteobacteria.Alphaproteobacteria.RF32_f_	3.26	Bacteria.Tenericutes.Mollicutes.RF39_f_g_s_	3.70
					Bacteria.Proteobacteria.Alphaproteobacteria.RF32_f_g_	3.15		
					Bacteria.Proteobacteria.Alphaproteobacteria.RF32_f_g_s_	3.14		
CPEAE v RREAE			Bacteria.Firmicutes.Bacilli.Turicibacterales.Turicibacteraceae	3.46			Bacteria.Bacteroidetes.Bacteroidia.Bacteroidales._Odoribacteraceae_	3.64
			Bacteria.Firmicutes.Bacilli.Turicibacterales.Turicibacteraceae.Turicibacter	3.46			Bacteria.Bacteroidetes.Bacteroidia.Bacteroidales._Odoribacteraceae_.Odoribacter	3.63
			Bacteria.Firmicutes.Bacilli.Turicibacterales.Turicibacteraceae.Turicibacter_s_	3.47			Bacteria.Bacteroidetes.Bacteroidia.Bacteroidales._Odoribacteraceae_.Odoribacter_s_	3.65
			Bacteria.Firmicutes.Clostridia.Clostridiales.Clostridiaceae.Clostridium	3.42			Bacteria.Bacteroidetes.Bacteroidia.Bacteroidales.Bacteroidaceae	4.43
			Bacteria.Firmicutes.Clostridia.Clostridiales.Clostridiaceae.Clostridium_s_	3.42			Bacteria.Bacteroidetes.Bacteroidia.Bacteroidales.Bacteroidaceae.Bacteroides	4.44
			Bacteria.Firmicutes.Clostridia.Clostridiales.Lachnospiraceae.rc4_4	3.71			Bacteria.Bacteroidetes.Bacteroidia.Bacteroidales.Bacteroidaceae.Bacteroides.acidifaciens	4.28
			Bacteria.Firmicutes.Clostridia.Clostridiales.Lachnospiraceae.rc4_4_s_	3.71			Bacteria.Bacteroidetes.Bacteroidia.Bacteroidales.Bacteroidaceae.Bacteroides_s_	3.89
			Bacteria.Firmicutes.Clostridia.Clostridiales.Peptococcaceae	3.71			Bacteria.Bacteroidetes.Bacteroidia.Bacteroidales.Porphyromonadaceae	3.72
			Bacteria.Firmicutes.Clostridia.Clostridiales.Ruminococcaceae.Ruminococcus	3.71			Bacteria.Bacteroidetes.Bacteroidia.Bacteroidales.Porphyromonadaceae.Parabacteroides	3.72
			Bacteria.Firmicutes.Clostridia.Clostridiales.Ruminococcaceae.Ruminococcus_s_	3.70			Bacteria.Bacteroidetes.Bacteroidia.Bacteroidales.Porphyromonadaceae.Parabacteroides_s_	3.71
			Bacteria.Verrucomicrobia	4.70			Bacteria.Bacteroidetes.Bacteroidia.Bacteroidales.Prevotellaceae	4.34
			Bacteria.Verrucomicrobia.Verrucomicrobiae	4.70			Bacteria.Bacteroidetes.Bacteroidia.Bacteroidales.Prevotellaceae.Prevotella	4.34
			Bacteria.Verrucomicrobia.Verrucomicrobiae.Verrucomicrobiales	4.70			Bacteria.Bacteroidetes.Bacteroidia.Bacteroidales.Prevotellaceae.Prevotella_s_	4.36
			Bacteria.Verrucomicrobia.Verrucomicrobiae.Verrucomicrobiales.Verrucomicrobiaceae	4.70			Bacteria.Bacteroidetes.Bacteroidia.Bacteroidales.Rikenellaceae	3.87
			Bacteria.Verrucomicrobia.Verrucomicrobiae.Verrucomicrobiales.Verrucomicrobiaceae.Akkermansia	4.66			Bacteria.Bacteroidetes.Bacteroidia.Bacteroidales.Rikenellaceae_g_	3.90
			Bacteria.Verrucomicrobia.Verrucomicrobiae.Verrucomicrobiales.Verrucomicrobiaceae.Akkermansia.muciniphila	4.67			Bacteria.Bacteroidetes.Bacteroidia.Bacteroidales.Rikenellaceae_g_s_	3.89
							Bacteria.Firmicutes.Clostridia.Clostridiales.Lachnospiraceae.Dorea	3.33
							Bacteria.Firmicutes.Clostridia.Clostridiales.Lachnospiraceae.Dorea_s_	3.15
							Bacteria.Proteobacteria.Gammaproteobacteria.Enterobacteriales	3.84
							Bacteria.Proteobacteria.Gammaproteobacteria.Enterobacteriales.Enterobacteriaceae	3.79
							Bacteria.Proteobacteria.Gammaproteobacteria.Enterobacteriales.Enterobacteriaceae_g_	3.78
							Bacteria.Proteobacteria.Gammaproteobacteria.Enterobacteriales.Enterobacteriaceae_g_s_	3.82
B6N v SJLN	Bacteria.Bacteroidetes	5.29			Bacteria.Firmicutes	5.32		
	Bacteria.Bacteroidetes.Bacteroidia	5.33			Bacteria.Firmicutes.Clostridia	5.30		
	Bacteria.Bacteroidetes.Bacteroidia.Bacteroidales	5.32			Bacteria.Firmicutes.Clostridia.Clostridiales	5.28		
	Bacteria.Bacteroidetes.Bacteroidia.Bacteroidales.S24_7	5.28			Bacteria.Firmicutes.Clostridia.Clostridiales.Lachnospiraceae	4.07		
	Bacteria.Bacteroidetes.Bacteroidia.Bacteroidales.S24_7_g_	5.27			Bacteria.Firmicutes.Clostridia.Clostridiales.Lachnospiraceae.Coprococcus	3.24		
	Bacteria.Bacteroidetes.Bacteroidia.Bacteroidales.S24_7_g_s_	5.30			Bacteria.Firmicutes.Clostridia.Clostridiales.Lachnospiraceae.Coprococcus_s_	3.22		
	Bacteria.Firmicutes.Bacilli	3.44			Bacteria.Firmicutes.Clostridia.Clostridiales.Lachnospiraceae_g_	4.16		
	Bacteria.Firmicutes.Bacilli.Lactobacillales	3.39			Bacteria.Firmicutes.Clostridia.Clostridiales.Lachnospiraceae_g_s_	4.21		
	Bacteria.Firmicutes.Bacilli.Lactobacillales.Lactobacillaceae	3.39			Bacteria.Firmicutes.Clostridia.Clostridiales.Ruminococcaceae	4.65		
	Bacteria.Firmicutes.Bacilli.Lactobacillales.Lactobacillaceae.Lactobacillus	3.38			Bacteria.Firmicutes.Clostridia.Clostridiales.Ruminococcaceae.Anaerotruncus	3.98		
	Bacteria.Firmicutes.Bacilli.Lactobacillales.Lactobacillaceae.Lactobacillus_s_	3.39			Bacteria.Firmicutes.Clostridia.Clostridiales.Ruminococcaceae.Anaerotruncus_s_	3.94		
	Bacteria.Firmicutes.Clostridia.Clostridiales._Mogibacteriaceae_	3.43			Bacteria.Firmicutes.Clostridia.Clostridiales.Ruminococcaceae.Oscillospira	4.44		
	Bacteria.Firmicutes.Clostridia.Clostridiales._Mogibacteriaceae__g_	3.26			Bacteria.Firmicutes.Clostridia.Clostridiales.Ruminococcaceae.Oscillospira_s_	4.47		
	Bacteria.Firmicutes.Clostridia.Clostridiales._Mogibacteriaceae__g_s_	3.16			Bacteria.Firmicutes.Clostridia.Clostridiales.Ruminococcaceae.Ruminococcus	3.70		
	Bacteria.Firmicutes.Clostridia.Clostridiales.Lachnospiraceae.Anaerostipes	3.42			Bacteria.Firmicutes.Clostridia.Clostridiales.Ruminococcaceae.Ruminococcus_s_	3.71		
	Bacteria.Firmicutes.Clostridia.Clostridiales.Lachnospiraceae.Anaerostipes_s_	3.40			Bacteria.Firmicutes.Clostridia.Clostridiales.Ruminococcaceae_g_	4.24		
	Bacteria.Firmicutes.Clostridia.Clostridiales.Lachnospiraceae.Dorea	3.60			Bacteria.Firmicutes.Clostridia.Clostridiales.Ruminococcaceae_g_s_	4.21		
	Bacteria.Firmicutes.Clostridia.Clostridiales.Lachnospiraceae.Dorea_s_	3.58			Bacteria.Firmicutes.Clostridia.Clostridiales_f_	5.14		
	Bacteria.Firmicutes.Clostridia.Clostridiales.Lachnospiraceae.rc4_4	3.82			Bacteria.Firmicutes.Clostridia.Clostridiales_f_g_	5.15		
	Bacteria.Firmicutes.Clostridia.Clostridiales.Lachnospiraceae.rc4_4_s_	3.57			Bacteria.Firmicutes.Clostridia.Clostridiales_f_g_s_	5.13		
	Bacteria.Firmicutes.Clostridia.Clostridiales.Peptococcaceae	3.64			Bacteria.Proteobacteria.Alphaproteobacteria	3.85		
	Bacteria.Firmicutes.Erysipelotrichi.Erysipelotrichales.Erysipelotrichaceae.Allobaculum	3.88			Bacteria.Proteobacteria.Alphaproteobacteria.RF32	4.10		
	Bacteria.Firmicutes.Erysipelotrichi.Erysipelotrichales.Erysipelotrichaceae.Allobaculum_s_	3.89			Bacteria.Proteobacteria.Alphaproteobacteria.RF32_f_	4.08		
					Bacteria.Proteobacteria.Alphaproteobacteria.RF32_f_g_	4.02		
					Bacteria.Proteobacteria.Alphaproteobacteria.RF32_f_g_s_	4.05		
B6N v CPEAE	Bacteria.Actinobacteria	3.25	Bacteria.Firmicutes.Clostridia.Clostridiales.Lachnospiraceae.rc4_4	3.67				
	Bacteria.Actinobacteria.Coriobacteriia	3.30	Bacteria.Firmicutes.Clostridia.Clostridiales.Lachnospiraceae.rc4_4_s_	3.67				
	Bacteria.Actinobacteria.Coriobacteriia.Coriobacteriales	3.29	Bacteria.Firmicutes.Clostridia.Clostridiales.Peptococcaceae	3.67				
	Bacteria.Actinobacteria.Coriobacteriia.Coriobacteriales.Coriobacteriaceae	3.29	Bacteria.Firmicutes.Erysipelotrichi.Erysipelotrichales.Erysipelotrichaceae.Coprobacillus	4.11				
	Bacteria.Actinobacteria.Coriobacteriia.Coriobacteriales.Coriobacteriaceae.Adlercreutzia	3.30	Bacteria.Firmicutes.Erysipelotrichi.Erysipelotrichales.Erysipelotrichaceae.Coprobacillus_s_	4.14				
	Bacteria.Actinobacteria.Coriobacteriia.Coriobacteriales.Coriobacteriaceae.Adlercreutzia_s_	3.36	Bacteria.Tenericutes.Mollicutes.RF39	3.04				
	Bacteria.Firmicutes.Bacilli	3.42	Bacteria.Tenericutes.Mollicutes.RF39_f_	3.05				
	Bacteria.Firmicutes.Bacilli.Lactobacillales	3.47	Bacteria.Tenericutes.Mollicutes.RF39_f_g_	3.05				
	Bacteria.Firmicutes.Bacilli.Lactobacillales.Lactobacillaceae	3.47	Bacteria.Tenericutes.Mollicutes.RF39_f_g_s_	3.04				
	Bacteria.Firmicutes.Bacilli.Lactobacillales.Lactobacillaceae.Lactobacillus	3.44	Bacteria.Verrucomicrobia	4.65				
	Bacteria.Firmicutes.Bacilli.Lactobacillales.Lactobacillaceae.Lactobacillus_s_	3.42	Bacteria.Verrucomicrobia.Verrucomicrobiae	4.65				
	Bacteria.Firmicutes.Clostridia.Clostridiales.Clostridiaceae_g_	3.73	Bacteria.Verrucomicrobia.Verrucomicrobiae.Verrucomicrobiales	4.65				
	Bacteria.Firmicutes.Clostridia.Clostridiales.Clostridiaceaeg_s_	3.72	Bacteria.Verrucomicrobia.Verrucomicrobiae.Verrucomicrobiales.Verrucomicrobiaceae	4.65				
	Bacteria.Firmicutes.Clostridia.Clostridiales.Lachnospiraceae	4.36	Bacteria.Verrucomicrobia.Verrucomicrobiae.Verrucomicrobiales.Verrucomicrobiaceae.Akkermansia	4.65				
	Bacteria.Firmicutes.Clostridia.Clostridiales.Lachnospiraceae._Ruminococcus_gnavus	3.41	Bacteria.Verrucomicrobia.Verrucomicrobiae.Verrucomicrobiales.Verrucomicrobiaceae.Akkermansia.muciniphila	4.65				
	Bacteria.Firmicutes.Clostridia.Clostridiales.Lachnospiraceae.Coprococcus	3.30						
	Bacteria.Firmicutes.Clostridia.Clostridiales.Lachnospiraceae.Coprococcus_s_	3.28						
	Bacteria.Firmicutes.Clostridia.Clostridiales.Lachnospiraceae.Dorea	3.79						
	Bacteria.Firmicutes.Clostridia.Clostridiales.Lachnospiraceae.Dorea_s_	3.72						
	Bacteria.Firmicutes.Clostridia.Clostridiales.Lachnospiraceae_g_	4.20						
	Bacteria.Firmicutes.Clostridia.Clostridiales.Lachnospiraceae_g_s_	4.21						
SJLN v RREAE					Bacteria.Cyanobacteria.4C0d_2.YS2	4.11	Bacteria.Bacteroidetes	5.31
					Bacteria.Firmicutes	5.35	Bacteria.Bacteroidetes.Bacteroidia	5.28
					Bacteria.Firmicutes.Clostridia	5.28	Bacteria.Bacteroidetes.Bacteroidia.Bacteroidales	5.28
					Bacteria.Firmicutes.Clostridia.Clostridiales	5.33	Bacteria.Bacteroidetes.Bacteroidia.Bacteroidales.Bacteroidaceae	4.42
					Bacteria.Firmicutes.Clostridia.Clostridiales.Lachnospiraceae	4.48	Bacteria.Bacteroidetes.Bacteroidia.Bacteroidales.Bacteroidaceae.Bacteroides	4.44
					Bacteria.Firmicutes.Clostridia.Clostridiales.Lachnospiraceae_g_	4.44	Bacteria.Bacteroidetes.Bacteroidia.Bacteroidales.Bacteroidaceae.Bacteroides.acidifaciens	4.22
					Bacteria.Firmicutes.Clostridia.Clostridiales.Lachnospiraceae_g_s_	4.47	Bacteria.Bacteroidetes.Bacteroidia.Bacteroidales.Prevotellaceae	4.31
					Bacteria.Firmicutes.Clostridia.Clostridiales.Ruminococcaceae	4.77	Bacteria.Bacteroidetes.Bacteroidia.Bacteroidales.Prevotellaceae.Prevotella	4.33
					Bacteria.Firmicutes.Clostridia.Clostridiales.Ruminococcaceae.Oscillospira	4.53	Bacteria.Bacteroidetes.Bacteroidia.Bacteroidales.Prevotellaceae.Prevotella_s_	4.32
					Bacteria.Firmicutes.Clostridia.Clostridiales.Ruminococcaceae.Oscillospira_s_	4.54	Bacteria.Bacteroidetes.Bacteroidia.Bacteroidales.S24_7	5.18
					Bacteria.Firmicutes.Clostridia.Clostridiales.Ruminococcaceae_g_	4.27	Bacteria.Bacteroidetes.Bacteroidia.Bacteroidales.S24_7_g_	5.14
					Bacteria.Firmicutes.Clostridia.Clostridiales.Ruminococcaceae_g_s_	4.25	Bacteria.Bacteroidetes.Bacteroidia.Bacteroidales.S24_7_g_s_	5.15
					Bacteria.Firmicutes.Clostridia.Clostridiales_f_	5.12	Bacteria.TM7.TM7_3.CW040.F16_g_s_	4.25
					Bacteria.Firmicutes.Clostridia.Clostridiales_f_g_	5.15		
					Bacteria.Firmicutes.Clostridia.Clostridiales_f_g_s_	5.13		
					Bacteria.Proteobacteria.Alphaproteobacteria.RF32	4.31		
					Bacteria.Proteobacteria.Alphaproteobacteria.RF32_f_	4.22		
					Bacteria.Proteobacteria.Alphaproteobacteria.RF32_f_g_	4.24		
					Bacteria.Proteobacteria.Alphaproteobacteria.RF32_f_g_s_	4.17		

Supplemental Table S12. LEfSe Analysis Results. Each row indicates the groups included in the LEfSe analysis, while each column represents the group for which the indicated biomarkers were detected. Taxa identified as statistically significant and biologically relevant are included along with the Linear Discriminant Analysis (LDA) score, alpha values > 0.05 (Kruskal-Wallis test) and a logarithmic LDA score > 3.0 were used as thresholds. Bacterial taxa in red are those taxa that were detected for a given group in all LEfSe analysis performed.

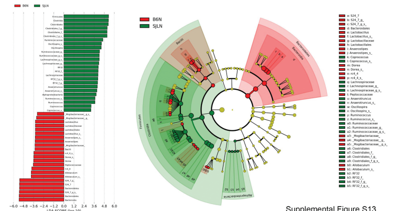

Supplemental Figure S13

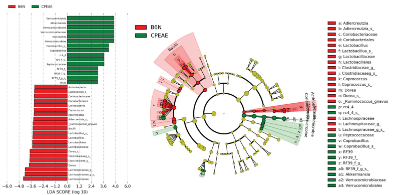

Supplemental Figure S14

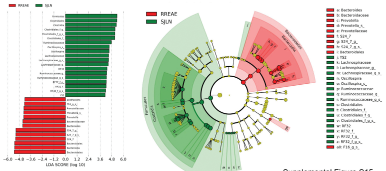

Supplemental Figure S15

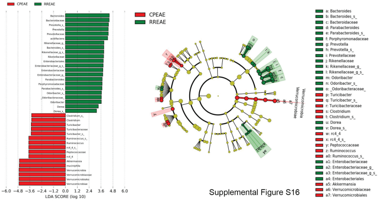

Supplemental Figure S16

Supplemental Figure Legends

Supplemental Figure S1. EAE disease course in chronic-progressive (CP-EAE) and relapse-remitting (RR-EAE) models of MS. A) Clinical scores were recorded daily following EAE induction by immunization with MOG_{35-55} or PLP $_{139-151}$ in C57BL/6 or SJL/J mice, respectively. The mean values shown are from a representative experiment with $\geq$ 10 mice per group. Average scores ± SEM are shown. Significance was assessed using Mann-Whitney U Test (*, p=0.0241). B) Chart representing change in mean disease score for RR-EAE mice, changes in ± 1 point indicate relapse (‡) or remittance (†).

Supplemental Figure S2. Clinical scores and encephalitogenic CD4+ T cell FoxP3 expression at time of sacrifice. A) Differences in disease scores between CP-EAE and RR-EAE mice at the time of sacrifice were assessed using Student's t-test (ns, p=0.772). B) Encephalitogenic CD4+ T-cells were isolated from CP-EAE and RR-EAE at the time of sacrifice and FoxP3 expression evaluated by qRT-PCR (n=3). Significance was assessed using a student's t-test (***, p=0.0002).

Supplemental Figure S10. Significantly different unclassified species. One-way ANOVA, followed by Tukey's multiple comparisons, was performed in order to assess significance. Bars represent mean ± SEM (adjusted p-values represented by * p<0.05; ** p<0.01; *** p<0.001; **** p<0.0001).

Supplemental Figure S13. LEfSe Analysis: biomarkers associated with naïve C57BL/6 and naïve SJL/J (A) A linear discriminant effect size (LEfSe) analysis was performed (alpha value $\geq$ 0.05, logarithmic LDA score threshold $\geq$ 3.0) on group pairs (B) The cladogram represents the phylogenetic relationship of significant OTUs associated with each group.

Supplemental Figure S14. LEfSe Analysis: biomarkers associated with naïve C57BL/6 and naïve CP-EAE (A) A linear discriminant effect size (LEfSe) analysis was performed (alpha value $\geq$ 0.05, logarithmic LDA score threshold $\geq$ 3.0) on group pairs (B) The cladogram represents the phylogenetic relationship of significant OTUs associated with each group.

Supplemental Figure S15. LEfSe Analysis: biomarkers associated with naïve SJL/J and RR-EAE (A) A linear discriminant effect size (LEfSe) analysis was performed (alpha value $\geq$ 0.05, logarithmic LDA score threshold $\geq$ 3.0) on group pairs (B) The cladogram represents the phylogenetic relationship of significant OTUs associated with each group.

Supplemental Figure S16. LEfSe Analysis: biomarkers associated with CP-EAE and RR-EAE (A) A linear discriminant effect size (LEfSe) analysis was performed (alpha value $\geq$ 0.05, logarithmic LDA score threshold $\geq$ 3.0) on group pairs (B) The cladogram represents the phylogenetic relationship of significant OTUs associated with each group.
